# LRP6 acts as a scaffold protein in cardiac gap junction assembly

**DOI:** 10.1038/ncomms11775

**Published:** 2016-06-02

**Authors:** Jun Li, Changming Li, Dandan Liang, Fei Lv, Tianyou Yuan, Erlinda The, Xiue Ma, Yahan Wu, Lixiao Zhen, Duanyang Xie, Shiyi Wang, Yuan Liu, Jian Huang, Jingyi Shi, Yi Liu, Dan Shi, Liang Xu, Li Lin, Luying Peng, Jianmin Cui, Weidong Zhu, Yi-Han Chen

**Affiliations:** 1 Key Laboratory of Arrhythmias of the Ministry of Education of China, East Hospital, Tongji University School of Medicine, Shanghai 200120, China; 2 Institute of Medical Genetics, Tongji University, Shanghai 200092, China; 3 Department of Cardiology, East Hospital, Tongji University School of Medicine, Shanghai 200120, China; 4 Cardiac Bioelectricity and Arrhythmia Center, and Department of Biomedical Engineering, Washington University, St Louis, Missouri 63130, USA; 5 Department of Pathology and Pathophysiology, Tongji University School of Medicine, Shanghai 200092, China

## Abstract

Low-density lipoprotein receptor-related protein 6 (LRP6) is a Wnt co-receptor in the canonical Wnt/β-catenin signalling. Here, we report the scaffold function of LRP6 in gap junction formation of cardiomyocytes. Cardiac LRP6 is spatially restricted to intercalated discs and binds to gap junction protein connexin 43 (Cx43). A deficiency in LRP6 disrupts Cx43 gap junction formation and thereby impairs the cell-to-cell coupling, which is independent of Wnt/β-catenin signalling. The defect in Cx43 gap junction resulting from LRP6 reduction is attributable to the defective traffic of *de novo* Cx43 proteins from the endoplasmic reticulum to the Golgi apparatus, leading to the lysosomal degradation of Cx43 proteins. Accordingly, the hearts of conditional cardiac-specific *Lrp6*-knockout mice consistently exhibit overt reduction of Cx43 gap junction plaques without any abnormality in Wnt signalling and are predisposed to lethal arrhythmias. These findings uncover a distinct role of LRP6 as a platform for intracellular protein trafficking.

## Abstract (web summary)

LRP6 is known for its role as a Wnt co-receptor essential for the canonical Wnt/β-catenin signaling. Here, Li *et al*. show that LRP6 exerts a Wnt-independent scaffold function and regulates connexin 43 gap junction formation and coupling of cardiomyocytes in mouse hearts.

## Graphical abstract

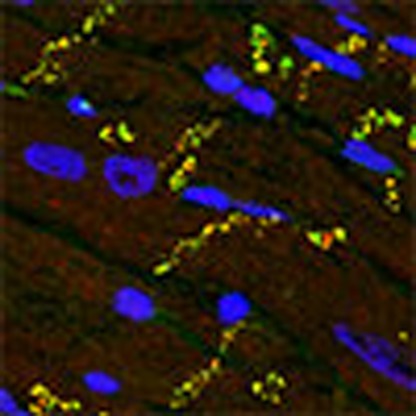

Wnts are secreted glycoproteins that activate evolutionarily conserved intracellular signalling cascades^1^. The Wnt signal has distinct transcriptional outputs, which determine cell proliferation, polarity and fate during embryonic development and tissue homeostasis^2^,^3^. The term Wnt is a combination of the Drosophila gene, *wingless*, and the vertebrate homologue, *Int-1* (ref. 1). As currently understood, Wnt proteins bind to frizzled receptors on the cell surface, and the resulting signal is transduced through several cytoplasmic relay components to β-catenin, which enters the nucleus and forms a complex with T-cell factor (TCF) to activate transcription of Wnt target genes^3^. For the interaction between Wnts and frizzled, co-receptors are commonly required^4^. The low-density lipoprotein receptor-related protein 6 (LRP6) is a documented co-receptor that mediates Wnt signalling dependent on β-catenin^4^,^5^.

Cumulative studies have demonstrated that participants in the Wnt signalling pathway play important roles in cardiovascular development and differentiation, angiogenesis, cardiac hypertrophy, cardiac failure and aging^6^,^7^,^8^,^9^,^10^,^11^. Given the central regulatory role of LRP6 in mediating the activation of the Wnt/β-catenin pathway^4^,^5^, germ-line knockout of *Lrp6* impairs Wnt signal transduction, thereby causing embryonic lethality in mice^12^. Mutations in the gene encoding LRP6 protein are implicated in human coronary artery disease^13^,^14^. However, additional new insights into the biological and physiological activities of LRP6 protein in the normal adult heart await further characterization.

In the present study, we uncovered a novel molecular property of LRP6, beyond its role as a Wnt co-receptor, in regulating connexin 43 (Cx43) gap junction-mediated intercellular communication. Reduction of LRP6 induced the retention of newly synthesized Cx43 in the endoplasmic reticulum (ER) and thereby promoted the lysosomal degradation of immature Cx43 proteins, which resulted in the impaired formation and function of gap junctions. Moreover, although LRP5 and LRP6 are highly homologous, the evidence obtained in this study excludes the potential contribution of LRP5 to Cx43 expression and the regulation of LRP6 on Cx43 gap junction. In summary, this study identified the novel scaffolding role of LRP6 in controlling intracellular Cx43 traffic and gap junction formation in the heart.

## Results

### LRP6 associates with Cx43 gap junction in cardiac myocytes

**Figure 1:**
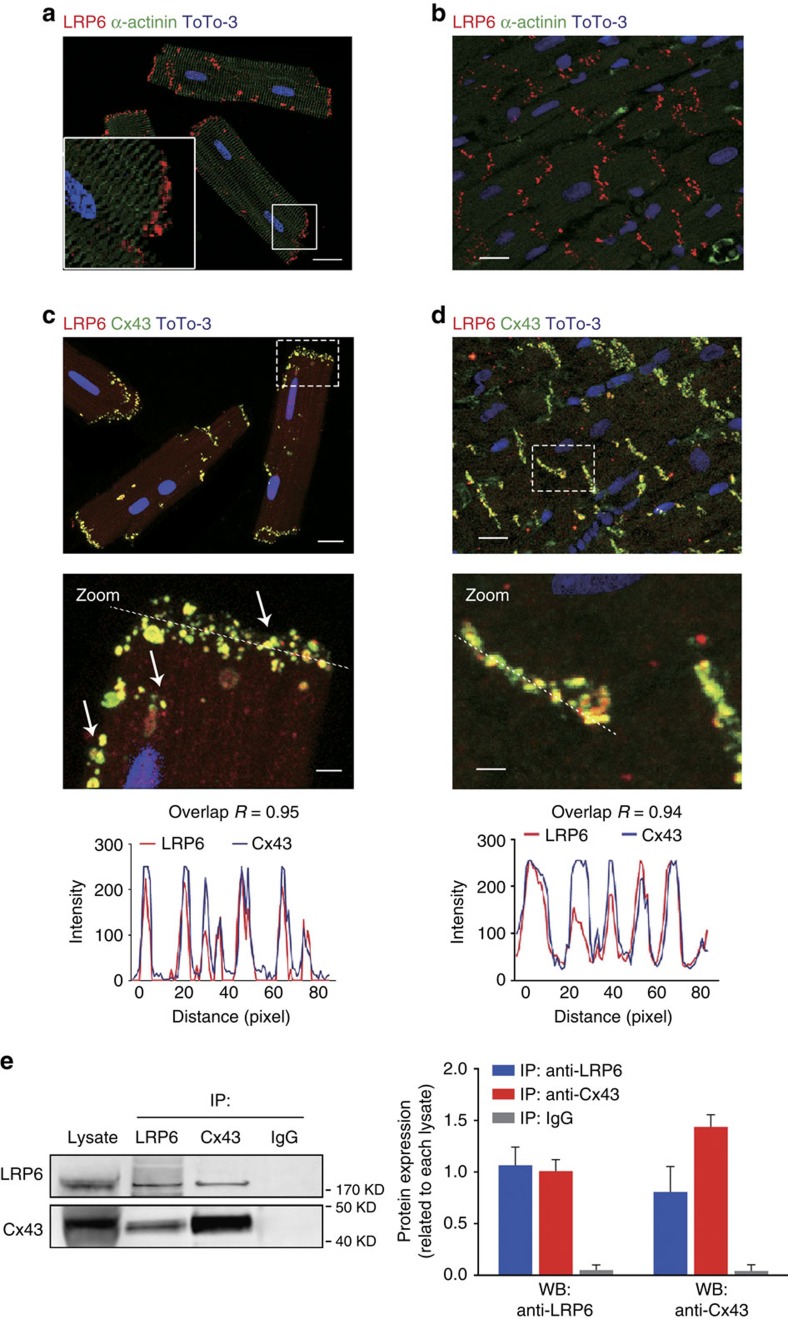
Spatial distribution of LRP6 in cardiomyocytes and ventricular tissues. (**a**,**b**) Immunofluorescence staining of endogenous LRP6 in intact adult mouse ventricular myocytes (AMVMs) (**a**) and intact adult mouse ventricular tissues (**b**). Scale bars, 25 μm. Magnified images indicates the distribution patterns of LRP6 in the white boxes. (**c**,**d**) Immunofluorescence labelling of endogenous LRP6 and Cx43 in intact AMVMs (**c**) and intact adult mouse ventricular tissues (**d**). Magnified images indicating co-localization of LRP6 with Cx43 are shown in the white boxes. Upper, scale bars, 25 μm; Middle, scale bars, 5 μm. The bottom row indicates Pearson's correlation coefficient (*R*) and line plot profiles. (**e**) Co-immunoprecipitation of endogenous LRP6 and Cx43 in intact AMVMs. Left, typical blots; right, pooled data. *n*=3. Data are means±s.e.m. *n* represents the number of experiments. The presented images and blots are representative of three separate experiments.

To analyse the cell biology of LRP6 in the heart, we first utilized confocal microscopy combined with immunofluorescence staining to visualize the cellular localization of LRP6. In freshly isolated, intact adult rat ventricular cardiomyocytes (Fig. 1a) and heart sections (Fig. 1b), LRP6 was predominantly stained at intercalated discs. Considering that gap junctions, principally composed of Cx43 proteins, are specialized intercellular connections between myocytes in the ventricle^15^,^16^, we next performed co-localization analysis of LRP6 and Cx43 in cardiomyocytes. The results showed nearly complete overlap of LRP6 with Cx43 (Fig. 1c,d). In accordance, LRP6 co-precipitated with Cx43 (Fig. 1e), which verified the physical interactions between endogenous LRP6 and Cx43. Interestingly, although LRP5 and LRP6 are transmembrane proteins whose large extracellular domains are highly related^5^, LRP5 did not interact with Cx43 (Supplementary Fig. 1a–e). Moreover, β-catenin, the key downstream effector of Wnt signalling^3^, was partially co-localized with Cx43 (Supplementary Fig. 1f). Collectively, the distinct cellular localization of LRP6 and the physical interaction of LRP6 and Cx43 suggest the potential regulation of Cx43 gap junctions in the heart by LRP6.

### Knockdown of LRP6 disrupts Cx43 gap junctions

**Figure 2:**
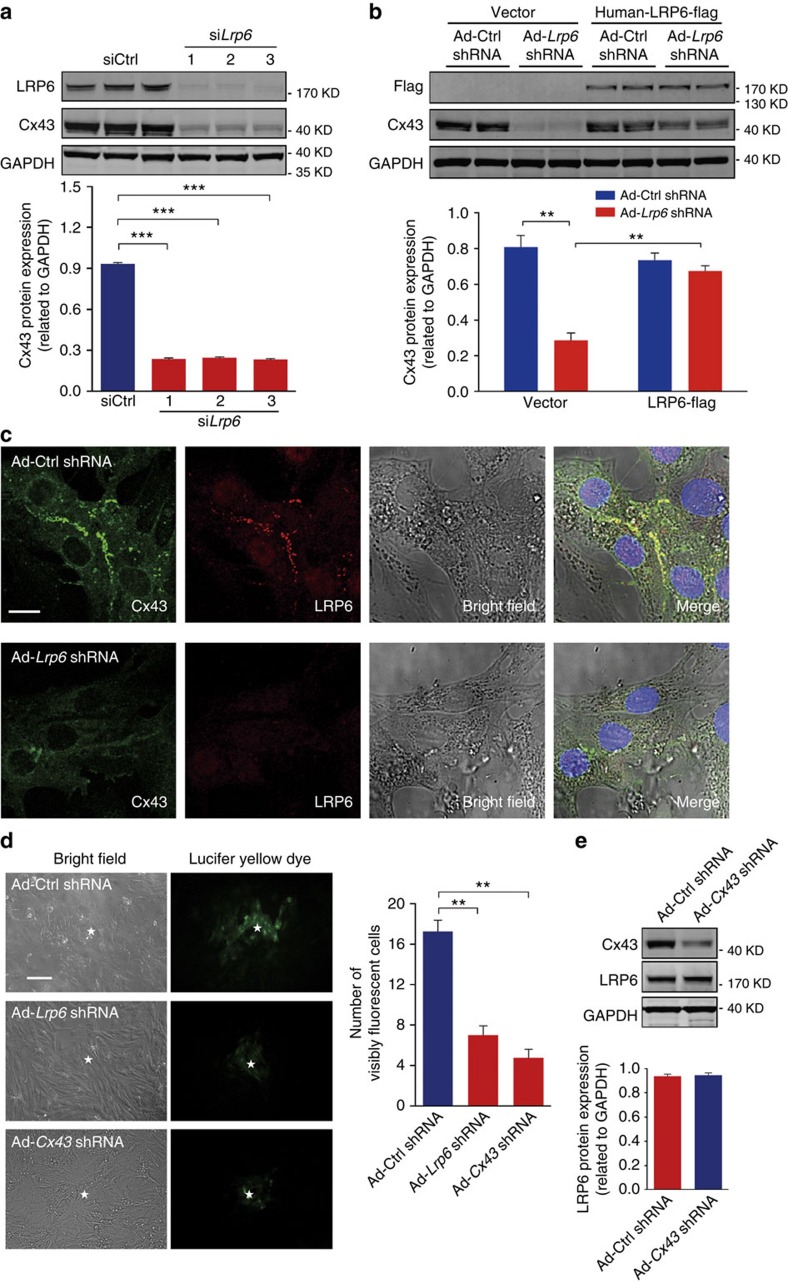
A deficiency of LRP6 depresses the expression and function of Cx43. (**a**,**b**) Effects of *Lrp6* knockdown on Cx43 protein levels in cultured NRVMs. Top, typical western blots; bottom, pooled data. Three independent *Lrp6* siRNA sequences were employed. *n*=3 (**a**,**b**). Data are means±s.e.m. ***P<0.001 compared with ctrl by one-way analysis of variance (ANOVA) with Bonferroni's *post hoc* test. *n* represents the number of experiments. (**c**) Immunofluorescence imaging of endogenous Cx43 gap junction in LRP6-deficient myocytes. Scale bar, 10 μm. The presented images are representative of three separate experiments. (**d**) Dye coupling in *Lrp6* knockdown, *Cx43* knockdown and control NRVMs. Photographs are taken at 1 min after Lucifer yellow dye injection and injected cardiomyocytes are indicated by asterisks. Scale bar, 10 μm. Note that *Lrp6* or *Cx43* knockdown significantly reduces dye coupling among cardiomyocytes. Left, typical images; right, pooled data. *n*=3. Data are means±s.e.m. **P<0.01 compared with ctrl by one-way ANOVA with Bonferroni's *post hoc* test. *n* represents the number of experiments. (**e**) Effects of *Cx43* knockdown on LRP6 protein expression in NRVMs. Top, typical blots; bottom, pooled data. *n*=3. Data are means±s.e.m. *n* represents the number of experiments.

To explore the potential effect of LRP6 on Cx43 gap junctions, *Lrp6* knockdown was conducted in cultured primary neonatal rat ventricular myocytes (NRVMs) using adenovirus-delivered small interfering RNAs (siRNAs) against the *Lrp6* transcript (Ad-*Lrp6* short hairpin RNA (shRNA)). The knockdown efficacies of three independent *Lrp6* siRNAs were preliminarily verified in NRVMs (Fig. 2a), and the most effective siRNA sequence was adopted to generate a high titre of Ad-*Lrp6* shRNA adenovirus. A reduction in LRP6 significantly depressed the protein expression of Cx43 (Fig. 2a), which was validated by overexpression of shRNA-resistant human *Lrp6* (Fig. 2b). LRP6 deficiency did not affect other proteins responsible for cell–cell junctions, including Cx40, Cx45 and N-cadherin (a transmembrane protein mediating cell–cell adhesion) (Supplementary Fig. 2a–d). Confocal fluorescence immunohistochemistry of NRVMs also confirmed the reduction in Cx43 protein levels and allowed for the visualization of the disruption of gap junction plaque formation in the presence of Ad-*Lrp6* shRNA (Fig. 2c). We next considered whether the reduction of Cx43 and the disruption of gap junction plaque by *Lrp6* knockdown have a functional effect on cell-to-cell coupling. Because gap junction channels composed of Cx43 are highly permeable to the fluorescent dye Lucifer Yellow, we tested the extent of intercellular transfer of the gap junction permeant Lucifer Yellow. As shown in Fig. 2d, the number of visibly fluorescent neighbour cells after injection into a centrally situated myocyte was significantly reduced in LRP6-deficient NRVMs, suggesting a defect in cell-to-cell coupling via Cx43 gap junctions. In contrast to the profound effects of *Lrp6* knockdown on Cx43 proteins, Cx43 reduction did not affect protein levels of LRP6 (Fig. 2e). It is worth noting that knockdown of *Lrp5* in NRVMs did not change Cx43 protein expression and gap junction formation, neither did LRP5 overexpression counteract the downregulation of Cx43 caused by LRP6 deficiency (Supplementary Fig. 3). These results indicate that LRP6 regulates the protein expression of Cx43, thereby affecting Cx43-dependent gap junction formation and function in cardiomyocytes.

LRP6 comprises four tandem β-propeller–epidermal growth factor-like domains (PE), LDLR type A domain (LA) and cytoplasmic domain (CD). To further identify the potentially key domains of LRP6 in regulating the protein expression of Cx43, we engaged multiple truncated mutants of LRP6 (Supplementary Fig. 4a). Overexpression of the truncated mutant with deletion of the first-to-second β-propeller–epidermal growth factor domain (ΔP1E1P2E2) rather than of the other modules (ΔP3E3P4E4 and ΔLA-CD) of LRP6 significantly downregulated the protein levels of Cx43 (Supplementary Fig. 4b), which was similar to the effect of silencing the full-length LRP6. In addition, we found that the LRP6-P1E1-P2E2 module was also responsible for the binding of LRP6 to Cx43 (Supplementary Fig. 4c), suggesting that the LRP6 P1E1-P2E2 domain is essential for the LRP6-dependent regulation of Cx43 protein expression.

### LRP6 regulates Cx43 expression independent of Wnt signalling

**Figure 3:**
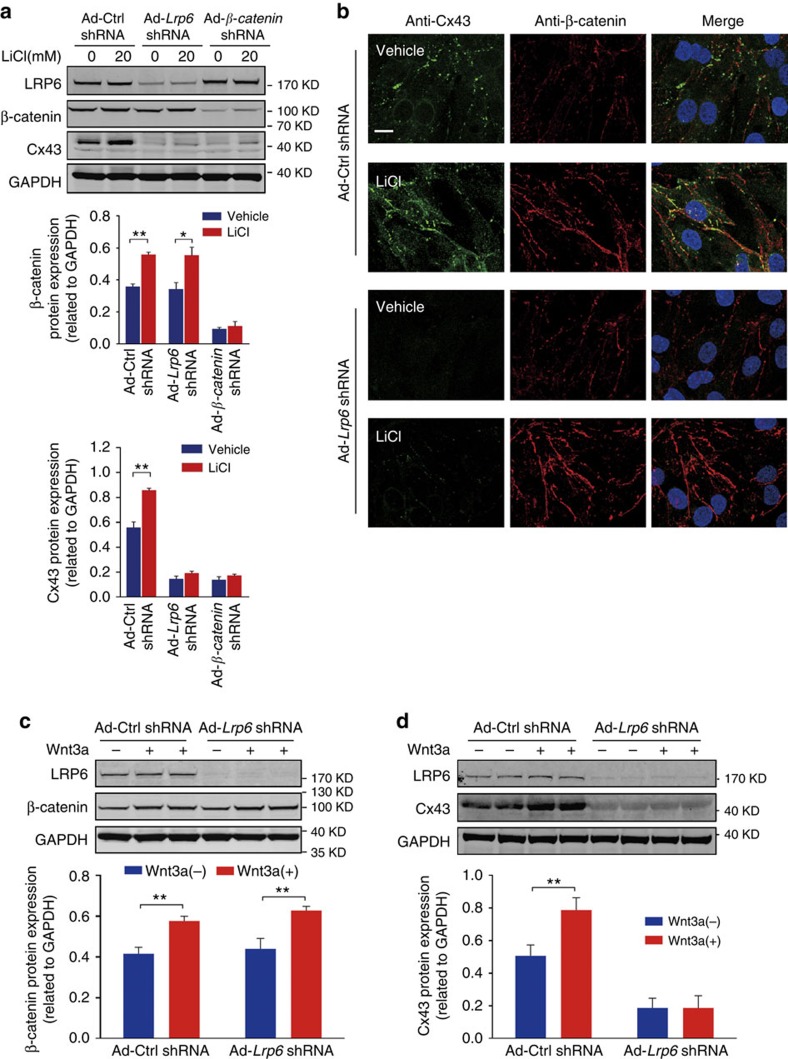
LRP6 regulates Cx43 expression independent of Wnt/β-catenin signalling. (**a**) Effects of LiCl-mediated Wnt activation on Cx43 protein in LRP6-deficient NRVMs. Upper, typical blots; middle and lower, pooled data. (**b**) Immunomicroscopic view of Cx43 in LRP6-deficient NRVMs exposed to LiCl (20 mM) for 24 h. Scale bar, 10 μm. The presented images are representative of three separate experiments. (**c**) Evaluation of Wnt signalling activation in LRP6-deficient NRVMs treated with Wnt3a. The Wnt3a-conditioned medium was adopted to treat NRVMs expressing Ad-*Lrp6* shRNA and the protein expression of β-catenin was examined. (**d**) Effects of Wnt signalling activation on Cx43 protein expression in LRP6-deficient NRVMs. Top, typical blots; bottom, pooled data. *n*=3 in (**a**,**c**,**d**). Data are means±s.e.m. *P<0.05 and **P<0.01 compared with ctrl by two-tailed unpaired Student's *t*-test. *n* represents the number of experiments.

Previous studies have suggested that Cx43 is a functional target for Wnt/β-catenin signalling^17^,^18^,^19^. Both Wnt ligands and lithium (an ion that mimics Wnt signalling) activate β-catenin to induce transcriptional activation of the *Cx43* promoter and increase Cx43 protein expression^18^,^19^. In the absence of a Wnt ligand, LRP6 is inactive; consequently, β-catenin is degraded via the proteasome^5^. We assessed whether LRP6 deficiency depressed the expression of β-catenin, and, if so, the potential association of this process with reduced Cx43 expression. Surprisingly, although the reduction in β-catenin reduced Cx43 protein levels (Fig. 3a), LRP6 deficiency did not alter the protein expression and cellular distribution of β-catenin in NRVMs (Supplementary Fig. 5a–c). In normal cardiomyocytes, Wnt activation and inhibition with Wnt3a and LGK-974 did not affect the membrane distribution and interaction of LRP6 and Cx43 (Supplementary Fig. 5d). On exposure to lithium chloride and Wnt3a-conditioned medium (Fig. 3a–c), the protein levels of β-catenin were upregulated in LRP6-deficient NRVMs; however, the reductions in Cx43 protein expression and gap junction formation were not ameliorated (Fig. 3d). These results indicate that LRP6 is not indispensable for Wnt/β-catenin signalling in the heart, and the regulation of Cx43 expression by LRP6 is independent of the Wnt/β-catenin signalling pathway.

### LRP6 modulates *Cx43* expression post-transcriptionally

**Figure 4:**
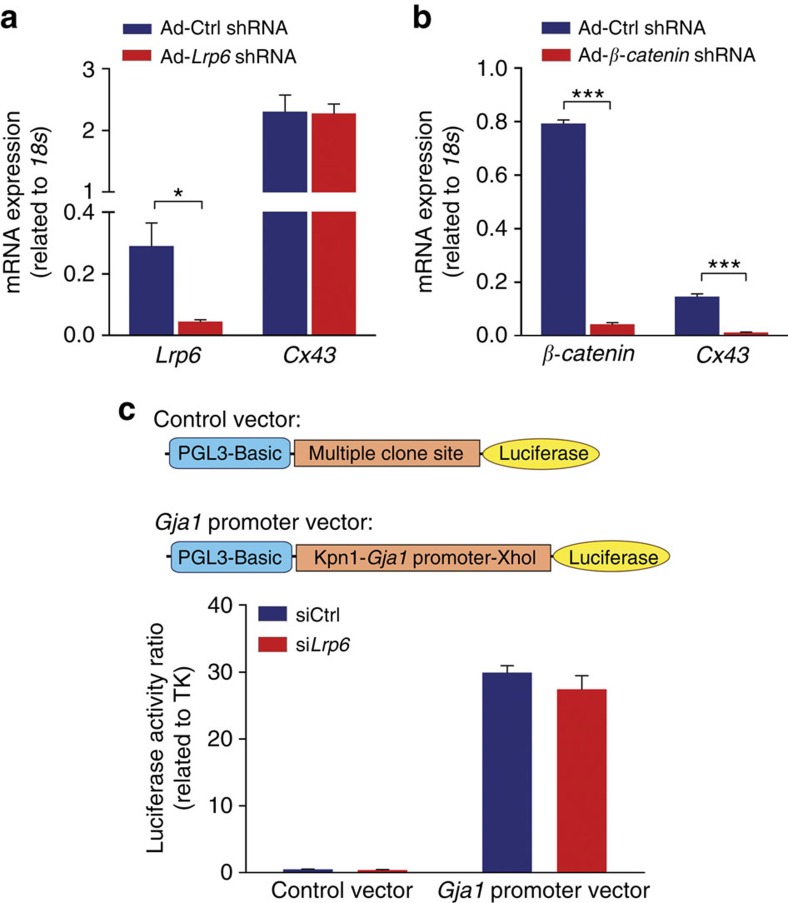
LRP6 modulates Cx43 expression post-transcriptionally. (**a**) Quantitative PCR analysis of *Cx43* transcript levels in *Lrp6*-knockdown NRVMs. *P<0.05 compared with ctrl by two-tailed unpaired Student's *t*-test. (**b**) Effects of *β-catenin* knockdown on *Cx43* mRNA expression. ***P<0.001 compared with ctrl by two-tailed unpaired Student's *t*-test. (**c**) Luciferase assay for the transcription of *Cx43*. Top, schematic diagram of the luciferase report vector containing the *Cx43* (*Gja1*) promoter. LRP6-deficient HEK 293 cells were transfected with a *Gja1* promoter–reporter gene construct for 24 h before activity measurement. *n*=3. Data are means±s.e.m. *n* represents the number of experiments.

To gain insight into the reduction of Cx43 protein levels caused by LRP6 loss-of-function, changes in *Cx43* transcription were investigated. Quantitative PCR analysis showed that LRP6 reduction did not change overall *Cx43* messenger RNA (mRNA) levels, in contrast to the decreased *Cx43* mRNA levels caused by *β-catenin* knockdown (Fig. 4a,b). By transfecting a *Cx43* promoter–reporter gene construct into human embryonic kidney 293 cells, we further measured the transcriptional activity of *Cx43* in the absence of LRP6. As shown in Fig. 4c, *Lrp6* knockdown did not have any effect on *Cx43* transcriptional activity. Collectively, the suppressive effect of LRP6 deficiency on the Cx43 protein was post-transcriptionally mediated.

### LRP6 reduction induces degradation of the ER-trapped Cx43

**Figure 5:**
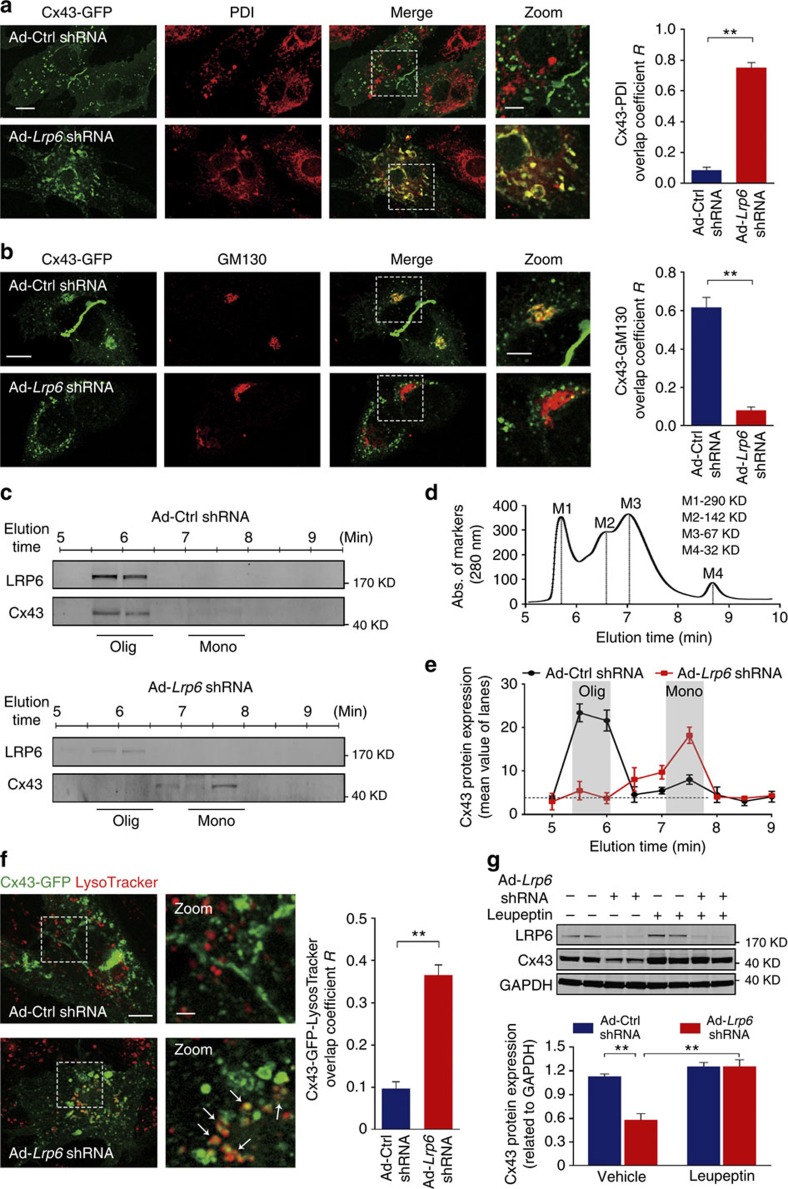
The ER-arrested Cx43 is degraded in lysosomes by *Lrp6* knockdown. (**a**,**b**) Immunofluorescence imaging of the co-localization of Cx43 with the ER and Golgi apparatus. PDI, an ER marker protein; GM130, a Golgi marker protein. Left, representative images; Scale bar, 10 μm. Right, analysis of overlap coefficient *R*. Scale bar for magnified images is 5 μm. The presented images are representative of three separate experiments. *n*=3 (**a**,**b**). Data are mean±s.e.m. **P<0.01 compared with ctrl by two-tailed unpaired Student's *t*-test. *n* represents the number of experiments. (**c**) The fractionated samples by gel filtration chromatography were analysed using western blot analysis with SDS-PAGE. (**d**,**e**) Gel filtration chromatography separation of cardiomyocyte proteins. (**d**) Separation of standard proteins (M1-M4). M1, glutamine dehydrogenase, 290 KD; M2, lactate dehydrogenase, 142 KD; M3, enolase, 67KD; M4, adenylate kinase, 32KD. (**e**) Protein expression analysis of **c**. Olig, oligomers; Mono, monomers. *n*=3. Data are means±s.e.m. *n* represents the number of experiments. (**f**) Imaging for co-localization analysis of Cx43 and lysosomes in NRVMs expressing Cx43-GFP. White arrows indicated the co-localization of Cx43 and lysosomes. Right, quantative analysis of Cx43-lysosome overlap. The presented images are representative of three separate experiments. Scale bar, 10 μm; Scale bar for magnified images is 3 μm. *n*=3. Data are means±s.e.m. **P<0.01 compared with ctrl by two-tailed unpaired Student's *t*-test. *n* represents the number of experiments. (**g**) Western blotting analysis of the effects of lysosomal inhibition on Cx43 protein expression in LRP6-deficient NRVMs. leupeptin (Leu) was adopted to inhibit lysosomal degradation. *n*=3. Data are means±s.e.m. **P<0.01 compared with ctrl by one-way analysis of variance (ANOVA) with Bonferroni's *post hoc* test. *n* represents the number of experiments.

The rapid turnover and specific plasma-membrane localization of Cx43 indicate that the intracellular movements of Cx43 proteins are highly regulated^20^. During the process of Cx43 protein turnover, nascent Cx43 proteins synthesized in the ER must be transported through the ER–Golgi network and intracellular transporting microtubules to their final destinations^20^,^21^,^22^,^23^. We monitored the dynamic trafficking of Cx43 using HeLa cells transfected with Cx43-green fluorescent protein (GFP). As shown in Supplementary Fig. 6a,b, the co-localization of LRP6 and Cx43 was most extensive in the ER at 5-h post transfection, and later on, their co-localization was mostly observed at the Golgi apparatus and along the microtubules (Supplementary Fig. 6b), suggesting that LRP6 constantly escorts Cx43 transport at the ER–Golgi interface and through the microtubule network to gap junction regions. Next, we assessed whether perturbed ER export contributed to Cx43 reduction in LRP6-deficient cardiomyocytes. As shown in Fig. 5a, in normal NRVMs transfected with Cx43-GFP, cell imaging displayed overt gap junction formation with very rare co-localization of Cx43 and PDI (an ER marker) at 24 h post transfection. In contrast, defective gap junctions and a significant overlap of Cx43 with PDI were observed in LRP6-deficient myocytes expressing Cx43-GFP. Notably, Cx43 strongly co-localized with GM130 in normal cardiomyocytes but not in LRP6-deficient cells (Fig. 5b). Despite the effect on Cx43 trafficking, *Lrp6* knockdown did not significantly affect the ER and Golgi network (Fig. 5a,b).

Physiologically, Cx43 proteins exit the ER as monomers and subsequently oligomerize in the Golgi complex^20^,^23^. To further verify the LRP6 modulation on Cx43 trafficking through the ER–Golgi network, we analysed the oligomerization of Cx43 using gel filtration chromatography to distinguish connexin monomers from oligomers. The Cx43 expressed in normal NRVMs was resolved predominantly as two major fractions with sizes corresponding to monomeric and oligomerized Cx43. *Lrp6* knockdown enriched the amount of Cx43 in the monomer band but, remarkably, reduced it in the oligomer band (Fig. 5c–e). These results indicate that LRP6 deficiency prevented the efficient trafficking of monomeric Cx43 from the ER to the Golgi apparatus and reduced the membrane expression of Cx43 proteins.

Cx43 proteins are short-lived and degrade through proteasomal/lysosomal pathways, whether Cx43 proteins are present in the secretory pathway (nascent connexins) or in gap junction plaques (mature connexins)^24^,^25^. The immature Cx43 monomers detained in the ER eventually enter the degradative pathway^26^. We then examined whether aberrant Cx43 protein degradation contributed to Cx43 protein reduction in LRP6-deficient cardiomyocytes. In NRVMs expressing Cx43-GFP, knockdown of *Lrp6* facilitated the entry of Cx43 into the lysosomes (Fig. 5f). Treatment of LRP6-deficient NRVMs with leupeptin (a lysosomal protease inhibitor) greatly counteracted the reduction in Cx43 protein levels (Fig. 5g), which was similar to the effects of lysosomal-associated membrane protein-1 (*Lamp1*) siRNA and Bafilomycin A1 (Ba-A1, an inhibitor of the late phase of autophagy; Supplementary Fig. 7a,b), whereas MG132 (a protease inhibitor) had no effect (Supplementary Fig. 7c,d). Moreover, we examined whether the reduced Cx43 by LRP6 deficiency was linked to the morphological and functional change in the ER. As shown in the Supplementary Fig. 8, neither the ER network nor the molecules for ER stress and protein exit machinery were affected. Taken together, LRP6 deficiency induces diversion of immature Cx43 proteins at the ER into lysosomal degradation, leading to Cx43 reduction.

### Lack of LRP6 in cardiomyocytes predisposes to arrhythmia

**Figure 6:**
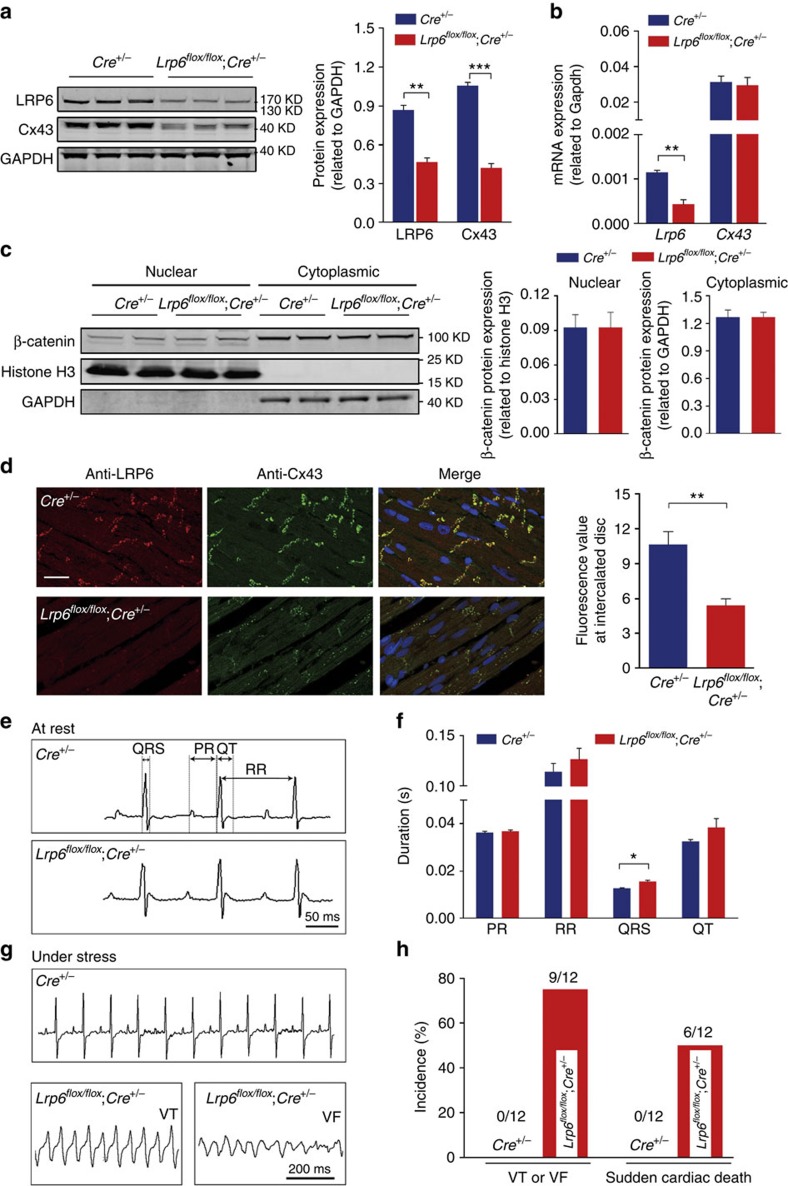
Conditional cardiac-specific *Lrp6*-knockout impairs cardiac gap junctions. (**a**,**b**) Immunoblotting and quantitative PCR measurement of *Lrp6* and *Cx43* in *Lrp6*-knockout mice hearts. The presented blots (**a**) and PCR results (**b**) were obtained from five *Lrp6*-knockout mice and three *Cre* transgene animals. Data are means±s.e.m. **P<0.01 and ***P<0.001 compared with *Cre*^*+/−*^ by two-tailed unpaired Student's *t*-test. (**c**) Cell fractional analysis of β-catenin protein in *Lrp6*-knockout mice hearts. Histone H3, a nuclear protein marker. Left, typical blots; right, pooled data. *n*=3. Data are means±s.e.m. *n* represents the number of experiments. (**d**) Immunofluorescence staining of Cx43 and LRP6 in ventricular myocardium sections of cardiac-specific *Lrp6*-knockout mice and *Cre* transgene controls. Left, typical images; right, quantification of Cx43 expression at intercalated discs in five *Lrp6*-knockout mice and three *Cre* transgene animals. Scale bar, 25 μm. Data are means±s.e.m. **P<0.01 compared with *Cre*^*+/−*^ by two-tailed unpaired Student's *t*-test. (**e**,**f**) Analysis of resting ECGs parameters through a telemetric ECG recording system in conscious mice. Left, representative ECGs; right, pooled data. The presented ECGs (**e**) and statistical analysis (**f**) were obtained from twelve *Lrp6*-knockout mice and twelve *Cre* transgene animals. Data are means±s.e.m. *P<0.05 compared with *Cre*^*+/−*^ by two-tailed unpaired Student's *t*-test. (**g**) Representative ECG recordings of conscious cardiac-specific *Lrp6*-knockout mice and *Cre* transgene controls under stress conditions. VT, ventricular tachycardia; VF, ventricular fibrillation. (**h**) Statistical analysis of sudden cardiac deaths and VT/VF incidence.

To investigate whether LRP6 deficiency was also sufficient to downregulate Cx43 in myocardium tissue *in vivo*, we crossed mice bearing the *Lrp6*^*flox/flox*^allele with a transgenic line (*MerCreMer*), which expresses *Cre* recombinase under the control of the α-myosin heavy chain promoter in a tamoxifen-inducible cardiomyocyte-specific manner, to produce the conditional cardiac-specific *Lrp6*-knockout (*Lrp6* CKO) mice (*Lrp6*^*flox/flox*^; *Cre*^+/−^ mice). To inactivate the LRP6 gene specifically in the heart, *Lrp6*^*flox/flox*^ mice with the Cre transgene were intraperitoneally administered tamoxifen (at E9.5 with a bolus injection and at 8 weeks old for 5 consecutive days, respectively). The *Lrp6*-CKO embryos demonstrated ventricular septal defect (Supplementary Fig. 9a), but the adult *Lrp6*-CKO animals appeared healthy and the *Lrp6*-CKO hearts were morphologically and functionally normal, as determined by histological analysis (Supplementary Fig. 9b,c) and echocardiographic measurements (Supplementary Fig. 9d). Histological analysis demonstrated that fibroblasts were interspersed throughout the myocardium and were accompanied by a small amount of collagen deposition (Supplementary Fig. 9c) in the *Lrp6*-CKO mice. Western blotting analyses of heart lysates revealed significant decreases in LRP6 and Cx43 proteins in *Lrp6*-CKO hearts compared with *Cre* transgene mice hearts (Fig. 6a). Quantitative PCR confirmed the reduction in *Lrp6* mRNA transcripts, which had no effect on the mRNA expression of *β-catenin* and *Cx43* (Fig. 6b). *Lrp6*-CKO did not change the nuclear localization of β-catenin (Fig. 6c). Immunofluorescence analyses of heart sections demonstrated significant reduction in Cx43 protein staining in the *Lrp6*-CKO hearts (Fig. 6d). A telemetric electrocardiograph recording of conscious *Lrp6*-CKO mice and *Cre* transgene mice revealed no significant difference in PR interval, heart rate and QT interval, but the duration of ventricular depolarization (QRS complex) was mildly prolonged in *Lrp6*-CKO mice (Fig. 6e,f). Moreover, the *Lrp6*-CKO mice between 6 and 8 weeks after tamoxifen administration were highly susceptible to ventricular tachycardia or fibrillation (75%) and sudden cardiac death (50%) when they were subjected to a stress-inducing protocol; conversely, no severe arrhythmia events were observed as mentioned above in *Cre* transgene control mice (Fig. 6g,h). These results suggest that cardiac *Lrp6*-knockout *in vivo* leads to marked downregulation of Cx43 gap junctions in intact hearts independently of Wnt signalling and consequently predisposes the hearts to lethal arrhythmias.

## Discussion

The findings reported here elucidate the unanticipated Wnt/β-catenin-independent scaffold function of LRP6 in regulating gap junctional coupling between individual cardiomyocytes. First, we identified the specific distribution of cardiac LRP6 within intercalated discs and the physical link between LRP6 and gap junction protein Cx43. Second, we revealed that LRP6 deficiency disrupts gap junction formation and function by impairing dynamics of Cx43 protein trafficking and stability. Third, we demonstrated that LRP6 reduction does not affect β-catenin protein expression in the presence or absence of a Wnt ligand. Fourth, the LRP6-dependent modulation of Cx43 is independent of β-catenin. Additionally, *Lrp6*-ablated mouse hearts showed defective Cx43 gap junctions but normal Wnt signalling and cardiac architecture. Taken together, these findings provide the first *in vitro* and *in vivo* evidence implicating LRP6 as an important platform for intracellular protein trafficking.

The biological significance of LRP6 has gained increasing recognition over the past decade. To date, our understanding of this protein is mostly limited to its critical roles in transducing canonical Wnt signalling^4^,^5^. LRP6-mediated Wnt signalling widely contributes to cell and tissue homeostasis^5^,^12^, and orients stem-cell division that determines distinct cell fate and organogenesis^5^,^27^. Alterations in the genes encoding LRP6 proteins disrupt the Wnt/β-catenin signalling pathway and consequent cell behaviours^5^. Unexpectedly, LRP6 is not necessary for Wnt signalling transduction in the heart, but it critically governs the formation and function of Cx43 gap junctions independent of Wnt/β-catenin signalling (Figs 2 and 3). Intriguingly, although LRP5 and LRP6 are highly homologous proteins with key functions in canonical Wnt signalling, LRP5 does not contribute to the regulation of Cx43 gap junction (Supplementary Figs 1 and 3), further supporting the distinct biological property of LRP6 in Cx43-mediated intercellular communication.

Cx43 molecules constitute the integral gap junction channel. The short half-life and rapid turnover kinetics of Cx43 implicate an important mechanism for degradative regulation of intercellular coupling within the heart^24^,^25^. In the present study, we observed a significant reduction in Cx43 protein levels when LRP6 was absent (Figs 2, 3 and 6). Similar to previous reports, which demonstrate that premature Cx43 in the ER is diverted from the secretory pathway to the degradation pathway^28^,^29^, we have characterized the defective transport of Cx43 from the ER to the Golgi apparatus and the pre-Golgi lysosomal degradation of newly synthesized Cx43 (Fig. 5), and our findings are in line with the concept that protein degradation by the ER occurs in the cytosol and lysosomes^26^. The LRP6-dependent modulation of Cx43 appears not to be involved in *Cx43* transcription, or movement along microtubules, or internalization from the membrane surface (Fig. 4 and Supplementary Figs 6, 10 and 11), which differs from β-catenin-mediated regulation of Cx43 (Fig. 4 and Supplementary Fig. 11). Thus, our findings lead to the theory that, in the process of gap junction formation, LRP6 may act as a novel scaffold protein for intracellular Cx43 trafficking.

Intercellular communication is part of a complex system of communication that governs basic cellular activities and coordinates cell actions^30^. Gap junction residing at cell–cell borders is one of the most common ways in which human cells communicate^30^. Its density is of general importance. Cx43 is a most widely expressed and abundant gap junction protein throughout different tissues and critically governs intercellular signalling communication^31^,^32^. The loss of Cx43 gap junctional communication leads to developmental defects and tumourigenesis^32^,^33^. In the heart, gap junctions join the ends of cardiomyocytes and ensure electric coupling between cells^34^. Following cardiac ischaemia, reduction of Cx43 gap junctions contributes to malignant arrhythmias^35^. Preventing or reversing this process offers a strategy to repair a damaged heart and ameliorate arrhythmogenesis^36^. Thus, the mechanistic characterization of LRP6-dependent regulation of Cx43 gap junction may have profound implications in development and diseases.

In conclusion, our findings identify a novel molecular property of LRP6 beyond its role as a Wnt co-receptor. The molecular identification of LRP6 as a regulator of gap junction formation in cardiomyocytes may provide novel insights into cardiac pathophysiology.

## Methods

This study conformed to the Guide for the Care and Use of Laboratory Animals published by the US National Institutes of Health (NIH Publication, 8th Edition, 2011) and the policy of the Animal Care and Use Committee of the Tongji University School of Medicine.

### Isolation of adult rat ventricular myocytes

Ventricular myocytes were isolated from the Langendorff-perfused hearts of adult Sprague Dawley rats (2 months old). Briefly, the heart was removed, mounted on the Langendorff apparatus and perfused with Ca^2+^-free Krebs–Henseleit bicarbonate buffer to wash out the blood. Then the heart was perfused with Krebs–Henseleit bicarbonate buffer containing 25 mg ml^−1^ of collagenase type II (Worthington) for 10 min for digestion. When the heart became flaccid, the atria and great vessels were removed, and the ventricle was cut into small pieces and filtered through a cell strainer (100 μm, BD Falcon). The flow-through cells were then pelleted by centrifugation at 530*g* for 3 min at room temperature and resuspended in Ca^2+^-free Tyrode's solution. The calcium concentration was then gradually increased to 1.2 mM over 60 min.

### Immunofluorescence

Cells were fixed with 4% ice-cold paraformaldehyde (PFA) (Sigma) for 15 min and permeabilized with 0.1% Triton X-100 (Sigma) in PBS (Gibco) for 10 min, followed by 30 min of blocking in 1% BSA (Roche). The cells were then stained overnight at 4 °C with the following primary antibodies: sarcomeric α-actinin (1:400; Sigma), LRP6 (1:100; Abcam), LRP5 (1:100; Abcam), Cx43 (1:200; Sigma), PDI (1:100; Novus), GM130 (1:100; CST), α-tubulin (1:400; Sigma), Cx45 (1:200; Abcam), Cx40 (1:200; Abcam) and β-catenin (1:100; Sigma). Next, the cells were washed with PBS and incubated for 1 h with the respective secondary antibodies conjugated to Alexa Fluor 488/555/633 (1:300; Invitrogen), after which the cells were stained with ToTo-3 (Invitrogen) for an additional 20 min if needed.

Sections of mouse and rat hearts were deparaffinized and rehydrated, and then boiled in sodium citrate solution for 20 min for antigen retrieval. Slides were processed for immunofluorescence visualization according to the procedure used for cultured cardiomyocytes. Images were captured using a Leica SP5 laser confocal microscope with a × 63 oil lens. Co-localization was analysed using Image-Pro Plus software (MediaCybernetics).

### Western blot and immunoprecipitation analysis

For western blot analysis, cells and tissue homogenates were lysed in a protein-lysis buffer containing 50 mM Tris-HCl, pH 7.5, 150 mM NaCl, 2 mM EDTA, 1% NP40, 1% Triton and protease inhibitors (Roche). The lysate supernatant was collected after centrifugation, run on 10% SDS–polyacrylamide gel electrophoresis (SDS-PAGE) gel (Invitrogen) and transferred to nitrocellulose membranes. The membranes were then incubated with the following primary antibodies: Cx43 (Sigma) at 1:8,000; LRP6 (CST) at 1:1,000; LRP5 (CST) at 1:1,000; β-catenin (Sigma) at 1:1,000; LAMP1 (Abcam) at 1:1,000; Flag (Sigma) at 1:1,000; Cx45 (Abcam) at 1:1,000; Cx40 (Abcam) at 1:1,000; N-cadherin (CST) at 1:1,000; Histone H3 (Abcam) at 1:1,000; p-IRE1α (CST) at 1:1,000; GRP78 (CST) at 1:1,000; CHOP (CST) at 1:1,000; Sar1a (Abcam) at 1:1,000; Sar1b (Abcam) at 1:1,000; DNM2 (Abcam) at 1:1,000 and GAPDH (CST) at 1:1,000. The secondary antibodies conjugated to infrared dyes (LI-COR Biosciences) were applied at a concentration of 1:10,000 and visualized using an Odyssey imager. Western Blots were quantified by densitometry using ImageJ software (NIH). Full scans of western blots are presented in Supplementary Fig. 12.

For co-immunoprecipitation analysis, protein lysate was incubated overnight at 4 °C with the primary antibodies against Cx43 (Sigma), LRP6 (CST), LRP5 (CST), Flag (Sigma) or isotype control immunoglobulin G (Sigma) at a concentration of 1:50 with constant stirring. The next day, 50 μl Dynabeads (Invitrogen) were added, and the mixture was incubated for 6 h at 4 °C with rotation. The bead–antibody complexes were collected. Next, the samples were analysed by regular western blot.

### Quantitative PCR

To assess the mRNA expression levels of *Lrp6*, *Cx43*, *Lrp5* and *β-catenin* in cells and heart tissues, RNA was extracted using TRI Reagent (Ambion) according to the manufacturer's protocol. complementary DNA was synthesized using the TaqMan Reverse Transcription Kit (Applied Biosystems). Quantitative PCR was then performed using the Power SYBR Green master mix (Applied Biosystems) on a 7,900 HT Real-Time PCR System (Applied Biosystems). The housekeeping gene *Gapdh* or *18 s* was used for normalization. The sequences of all PCR primers are listed in Supplementary Table 1.

### Isolation and transfection of neonatal rat ventricular myocytes

Ventricles from neonatal rats were separated from the atria, cut into small pieces (∼2 × 2 mm^2^) and then dissociated in Ca^2+^-free HBSS containing 0.125 mg ml^−1^ trypsin (Gibco), 10 μg ml^−1^ DNase II (Sigma) and 0.1 mg ml^−1^ collagenase type IV (Sigma). Digestion was performed at 37 °C by stirring the digestion solution containing the heart sections throughout the repeated 5-min period of digestion for 8–10 times. The supernatant was collected with FBS (Gibco) after each digestion period. After digestion, the collected supernatant was centrifuged at 600*g* for 10 min at room temperature, and then cell pellets were resuspended in DMEM (Gibco) supplemented with 10% FBS and with 100 μM 5-bromo-2′-deoxyuridine (Sigma). The resuspended cells were passed through a cell strainer (100 mm, BD Falcon) and seeded onto 100-mm plastic dishes for 2 h at 37 °C in a 5% CO_2_ and humidified atmosphere. The supernatant was then collected and plated onto 1% gelatin (Sigma)-coated dishes. Twenty-four hours after the seeding, the medium was changed to DMEM (Gibco) containing 2% FBS (Gibco), 1% insulin-transferrin-selenium (ITS; Gibco), 1% Penicillin–Streptomycin (Gibco) and 100 μM 5-bromo-2′-deoxyuridine (Sigma).

For some gene silencing studies, cardiomyocytes were infected with adenoviral vectors expressing shRNAs against *Lrp6*, *Cx43*, *Lrp5* or *β-catenin* at the MOI (multiplicity of infection) of 100 (Hanbio Co. Ltd, Shanghai, China) after 72 h of seeding. Meanwhile, siRNA against LAMP1 and three independent siRNAs against *Lrp5* or *Lrp6* were transfected using Lipofectamine RNAiMAX (Invitrogen) after 72 h of cardiomyocytes seeding. All shRNA and siRNA sequences are listed in Supplementary Tables 2 and 3. Twenty-four hours after the treatment of adenoviral shRNAs, Lrp6, Lrp5 and Cx43 were overexpressed by transfecting their respective plasmids using Lipofectamine 3,000 (Invitrogen).

### Plasmids and reagents

Plasmids expressing LRP6, LRP5 or Cx43 were purchased from Origene. LiCl, leupeptin, MG132, nocodazole and dynasore were all purchased from Sigma. Brefeldin A and LGK-974 were purchased from Selleck. Bafilomycin A1 was purchased from Sangon Biotech in Shanghai.

### Lucifer yellow dye transfer

Neonatal myocytes were cultured on 35-mm glass-bottom dishes. Individual myocytes were injected with Lucifer Yellow (4% wt/vol in 150 mM LiCl) via microelectrodes (20 MΩ when filled with KCl) using overcompensation of the negative capacitance circuit on an electrometer (World Precision Instruments, Sarasota, Florida, USA) until the cell glowed brightly (<1 min). Cells were viewed under a Leica microscope equipped with fluorescence illumination and FITC filters, and pictures were captured every 30 s over a period 2 min.

### Wnt3a treatment

The cell line stably expressing Wnt3a was a kind gift from Dr Xiaoqing Zhang (Tongji University School of Medicine, China). Cells were grown in a T75 flask at a density of 2 × 10^5^ per flask in DMEM containing 10% FBS (Gibco). When cells grew to 70% confluence, the medium was replaced by DMEM without serum and further cultured for another 2 days. The medium was then collected and centrifuged at 600*g* for 10 min, and the supernatant was stored at 4 °C. After myocytes were transfected using Ad-shRNAs for 24 h, the myocyte medium was replaced with the above pre-warmed wnt3a-conditioned medium every day. Myocytes were harvested after another 48 h of culture and analysed by western blot.

### Reporter gene assay

To construct a luciferase reporter plasmid for the *Cx43* promoter, a fragment containing the human *Gja1* promoter region was amplified from human genomic DNA by PCR using specific primers (F: 5′-GGGGTACCCAACTGACAAATTGCTTAT-3′; R: 5′-CCGCTCGAGGCTCTCAGACCTGTCTGT-3′) and inserted into the luciferase plasmid (pGL3). To examine the effect of LRP6 deficiency on the activity of the *Gja1* promoter, HEK 293 cells were transfected with si*Lrp6* or siCtrl, and, 24 h later, the *Gja1* promoter luciferase reporter plasmid was transfected using Lipofectamine 3,000 (Invitrogen). After another 24 h of incubation, luciferase activities were measured using a dual luciferase reporter assay system (Promega).

### Isolation of membrane fractions from myoctyes

Myocytes were washed twice with ice-cold PBS and harvested in physiological buffer (10 mM Tris-HCl (pH 7.4), 5 mM EDTA, 2 mM dithiothreitol (DTT), 1 mM phenylmethyl sulphonyl fluoride (PMSF), 2 μg ml^−1^ leupeptin, 2 μg ml^−1^ pepstatin A, 140 mM NaCl; 800 μl per 100-mm dish). The cells were homogenized via 30 strokes with a Dounce homogenizer and were then centrifuged at 500*g* at 4 °C for 10 min. The collected supernatant was further centrifuged at 100,000*g* for 60 min at 4 °C. The supernatant was the cytosolic fraction. The pellet was resuspended in PBS containing 1% Triton X-100, and the solubilized proteins were designated as the membrane fractions. Each fraction was analysed by SDS-PAGE.

### Isolation of nuclear fractions from myocytes

Myocytes were collected and centrifuged for 5 min at 450*g*. The cell pellet was resuspended in lysis buffer (10 mM Tris-HCl, pH 7.5, with 2 mM MgCl_2_, 3 mM CaCl_2_ and 0.3 M Sucrose, 1 mM DTT, 1 mM PMSF, 2 μg ml^−1^ leupeptin and 2 μg ml^−1^ pepstatin A). The cells were homogenized with five up-and-down strokes in a glass tissue homogenizer, and then centrifuged for 20 min at 10,000*g*. The supernatant is the cytoplasmic fraction. The crude nuclei pellet was resuspended in extraction buffer (20 mM HEPES, pH 7.9, with 1.5 mM MgCl_2_, 0.42 M NaCl, 0.2 mM EDTA, 25% (v/v) Glycerol and 1 mM DTT, 1 mM PMSF, 2 μg ml^−1^ leupeptin and 2 μg ml^−1^ pepstatin A), gently shaked for 30 min and centrifuged for 5 min at 20,000*g*. The supernatant is the final nuclear fraction. Each fraction was analysed by SDS-PAGE.

### Gel filtration chromatography

Gel filtration chromatography was used for determination of Cx43 monomers and oligomers. Myocytes were washed twice with ice-cold PBS and harvested in physiological buffer (10 mM Tris-HCl (pH 7.4), 5 mM EDTA, 2 mM DTT, 1 mM PMSF, 2 μg ml^−1^ leupeptin, 2 μg ml^−1^ pepstatin A, 140 mM NaCl; 800 μl per 100-mm dish). The cells were homogenized via 30 strokes with a Dounce homogenizer and were then centrifuged at 500*g* at 4 °C for 10 min. 1% Triton X-100 was added to solubilize the membrane protein. After 1 h of incubation, the sample was centrifuged in a Beckman TLA 100.3 rotor at 55,000 r.p.m. (100,000*g*) for 30 min at 4 °C. Before gel filtration chromatography, the TSK-GEL G2000SW_XL_ (TSK Company Ltd, Japan) column was equilibrated with 20 mM of phosphate buffer, pH 7.0. Then the supernatant obtained after centrifugation was filtered through a syringe filter with a pore size of 0.2 μm and applied to the column. Sample volumes were maintained at <0.5 ml. A flow rate of 1 ml min^−1^ was used. Fractions were collected every 30 s throughout a period of 12 min. Next, these fractions were analysed by 10% SDS-PAGE and immunoblotting.

### Generation of conditional cardiac-specific *Lrp6*-knockout mice

Previously generated α*MHC*/*MerCreMer* mice were mated with *Lrp6*^flox/flox^ mice (kindly gifted by Dr Bart O. Williams, Center for Skeletal Disease and Tumor Metastasis and Laboratory of Cell Signaling and Carcinogenesis, Van Andel Research Institute, Grand Rapids, Michigan, USA) to produce α*MHC*/*MerCreMer*; *Lrp6*^flox/flox^ animals. The mice were maintained with a mixed C57BL genetic background. They were treated with tamoxifen (Sigma) via intraperitoneal injection once a day for 5 consecutive days at a dosage of 80 mg per kg of body weight per day. To study the potential effects of LRP6 on embryonic heart development, the mice on pregnancy days 9.5 were intraperitoneally treated with tamoxifen via a bolus injection.

### ECG recording

Telemetric ambulatory long-term ECG recordings were obtained with implantable transmitters. Mice were anaesthetized with ketamine hydrochloride (100 mg kg^−1^, intraperitoneal injection (IP)) and pentobarbital (30 mg kg^−1^, IP), and a midline incision was made along the spine. An implantable 3.5-g wireless radiofrequency transmitter (DataSciences International) was aseptically inserted into a subcutaneous tissue pocket in the peritoneum. The mice were placed in cages overlying a receiver for transmission of ECG signals to a computer for display and analysis, including assessment of heart rate variability parameters. The inducible arrhythmic events were tested according to the stress protocol with epinephrine (2 mg kg^−1^, IP) plus caffeine (120 mg kg^−1^, IP) in both the knockout and age-matched *Cre* transgene littermates.

### Echocardiography analysis

To evaluate left ventricular function and dimension, transthoracic two-dimensional echocardiography was performed on mice sedated with 5% isoflurane using a Visual Sonics Vevo 770 ultrasound system (Visual Sonics) equipped with a 30-MHz linear array transducer. M-mode tracings in the parasternal short axis view were used to measure left ventricular anterior and posterior wall thicknesses and left ventricular internal diameters at end-systole and end-diastole, which were used to calculate left ventricular fractional shortening and the ejection fraction according to standard formulas.

### Heart collection and histological analysis

At the end of the above experiments, animals were anaesthetized with 5% isoflurane and then killed by injection with 10% KCl to stop the heart at diastole. The hearts were excised, briefly washed in PBS, weighed, fixed in 10% formalin at room temperature, embedded in paraffin and further processed for histology. Haematoxylin-eosin and Masson's trichrome staining were performed according to standard procedures, and samples were analysed for regular morphology and the extent of fibrosis.

### Quantification of Cx43 gap junction plaque

ImageJ software was used to subtract the background from each image, and the rolling ball radius was set at 10.00 pixels. For each sample, the fluorescence intensity of six randomly selected images containing ∼20 intercalated discs was analysed.

### Statistics

All data are presented as the means±s.e.m. Statistical analyses were performed using Prism Software (GraphPad). The statistical significance of the difference between two sets of data was assessed using an unpaired, two-tailed Student's *t*-test and one-way analysis of variance with Bonferroni's *post hoc* test. A *P* value <0.05 was considered to be significant.

### Data availability

All relevant data are available from the authors on request and are included with the manuscript.

## Additional information

**How to cite this article:** Li, J. *et al*. LRP6 acts as a scaffold protein in cardiac gap junction assembly. *Nat. Commun.* 7:11775 doi: 10.1038/ncomms11775 (2016).

## Supplementary Material

Supplementary Information Supplementary Figures 1 - 12 and Supplementary Tables 1 - 3

## References

[b1] Nusse R. & Varmus H. E. Wnt genes. Cell 69, 1073–1087 (1992). 1617723 10.1016/0092-8674(92)90630-u

[b2] Cadigan K. M. & Nusse R. Wnt signaling: a common theme in animal development. Genes Dev. 11, 3286–3305 (1997). 9407023 10.1101/gad.11.24.3286

[b3] Logan C. Y. & Nusse R. The Wnt signaling pathway in development and disease. Annu. Rev. Cell Dev. Biol. 20, 781–810 (2004). 15473860 10.1146/annurev.cellbio.20.010403.113126

[b4] Tamai K. . LDL-receptor-related proteins in Wnt signal transduction. Nature 407, 530–535 (2000). 11029007 10.1038/35035117

[b5] Joiner D. M., Ke J., Zhong Z., Xu H. E. & Williams B. O. LRP5 and LRP6 in development and disease. Trends Endocrinol. Metab. 24, 31–39 (2013). 23245947 10.1016/j.tem.2012.10.003 PMC3592934

[b6] Rao T. P. & Kühl M. An updated overview on Wnt signaling pathways: a prelude for more. Circ. Res. 106, 1798–1806 (2010). 20576942 10.1161/CIRCRESAHA.110.219840

[b7] Gessert S. & Kühl M. The multiple phases and faces of wnt signaling during cardiac differentiation and development. Circ. Res. 107, 186–199 (2010). 20651295 10.1161/CIRCRESAHA.110.221531

[b8] Zhou Y. . Canonical WNT signaling components in vascular development and barrier formation. J. Clin. Invest. 124, 3825–3846 (2014). 25083995 10.1172/JCI76431 PMC4151216

[b9] Bergmann M.W. WNT signaling in adult cardiac hypertrophy and remodeling: lessons learned from cardiac development. Circ. Res. 107, 1198–1208 (2010). 21071717 10.1161/CIRCRESAHA.110.223768

[b10] Malekar P. . Wnt signaling is critical for maladaptive cardiac hypertrophy and accelerates myocardial remodeling. Hypertension 55, 939–945 (2010). 20177000 10.1161/HYPERTENSIONAHA.109.141127

[b11] Naito A. T., Shiojima I. & Komuro I. Wnt signaling and aging-related heart disorders. Circ. Res. 107, 1295–1303 (2010). 21106946 10.1161/CIRCRESAHA.110.223776

[b12] Pinson K. I., Brennan J., Monkley S., Avery B. J. & Skarnes W. C. An LDL-receptor-related protein mediates Wnt signalling in mice. Nature 407, 535–538 (2000). 11029008 10.1038/35035124

[b13] Mani A. . LRP6 mutation in a family with early coronary disease and metabolic risk factors. Science 315, 1278–1282 (2007). 17332414 10.1126/science.1136370 PMC2945222

[b14] Xu Y. . Functional analysis LRP6 novel mutations in patients with coronary artery disease. PLoS ONE 9, e84345 (2014). 24427284 10.1371/journal.pone.0084345 PMC3888387

[b15] Severs N. J. The cardiac gap junction and intercalated disc. Int. J. Cardiol. 26, 137–173 (1990). 2406208 10.1016/0167-5273(90)90030-9

[b16] Van Kempen M. J., Fromaget C., Gros D., Moorman A. F. & Lamers W. H. Spatial distribution of connexin43, the major cardiac gap junction protein, in the developing and adult rat heart. Circ. Res. 68, 1638–1651 (1991). 1645233 10.1161/01.res.68.6.1638

[b17] Olson D. J., Christian J. L. & Moon R. T. Effect of wnt-1 and related proteins on gap junctional communication in Xenopus embryos. Science 252, 1173–1176 (1991). 2031187 10.1126/science.252.5009.1173

[b18] van der Heyden M. A. . Identification of connexin43 as a functional target for Wnt signalling. J. Cell Sci. 111, 1741–1749 (1998). 9601103 10.1242/jcs.111.12.1741

[b19] Ai Z., Fischer A., Spray D. C., Brown A. M. & Fishman G. I. Wnt-1 regulation of connexin43 in cardiac myocytes. J. Clin. Invest. 105, 161–171 (2000). 10642594 10.1172/JCI7798 PMC377428

[b20] Martin P. E., Blundell G., Ahmad S., Errington R. J. & Evans W. H. Multiple pathways in the trafficking and assembly of connexin 26, 32 and 43 into gap junction intercellular communication channels. J. Cell Sci. 114, 3845–3855 (2001). 11719551 10.1242/jcs.114.21.3845

[b21] Majoul I. V. . Limiting transport steps and novel interactions of Connexin-43 along the secretory pathway. Histochem. Cell Biol. 132, 263–280 (2009). 19626334 10.1007/s00418-009-0617-x PMC2756399

[b22] Giepmans B. N. . Gap junction protein connexin-43 interacts directly with microtubules. Curr. Biol. 11, 1364–1368 (2001). 11553331 10.1016/s0960-9822(01)00424-9

[b23] Lauf U. . Dynamic trafficking and delivery of connexons to the plasma membrane and accretion to gap junctions in living cells. Proc. Natl Acad. Sci. USA 99, 10446–10451 (2002). 12149451 10.1073/pnas.162055899 PMC124935

[b24] Saffitz J. E., Laing J. G. & Yamada K. A. Connexin expression and turnover: implications for cardiac excitability. Circ. Res. 86, 723–728 (2000). 10764404 10.1161/01.res.86.7.723

[b25] Smyth J. W. & Shaw R. M. The gap junction life cycle. Heart Rhythm 9, 151–153 (2012). 21798227 10.1016/j.hrthm.2011.07.028 PMC3210376

[b26] Lippincott-Schwartz J., Bonifacino J. S., Yuan L. C. & Klausner R. D. Degradation from the endoplasmic reticulum: disposing of newly synthesized proteins. Cell 54, 209–220 (1988). 3292055 10.1016/0092-8674(88)90553-3

[b27] Habib S. J. . A localized Wnt signal orients asymmetric stem cell division in vitro. Science 339, 1445–1448 (2013). 23520113 10.1126/science.1231077 PMC3966430

[b28] Das S. . ERp29 restricts Connexin43 oligomerization in the endoplasmic reticulum. Mol. Biol. Cell 20, 2593–2604 (2009). 19321666 10.1091/mbc.E08-07-0790 PMC2682600

[b29] Maza J., Das Sarma J. & Koval M. Defining a minimal motif required to prevent connexin oligomerization in the endoplasmic reticulum. J. Biol. Chem. 280, 21115–21121 (2005). 15817491 10.1074/jbc.M412612200

[b30] Ruch R. J. Intercellular communication, homeostasis, and toxicology. Toxicol. Sci. 68, 265–266 (2002). 12151619 10.1093/toxsci/68.2.265

[b31] Naus C. C. & Laird D. W. Implications and challenges of connexin connections to cancer. Nat. Rev. Cancer 10, 435–441 (2010). 20495577 10.1038/nrc2841

[b32] Kameritsch P., Khandoga N., Pohl U. & Pogoda K. Gap junctional communication promotes apoptosis in a connexin-type-dependent manner. Cell Death Dis. 4, e584 (2013). 23579271 10.1038/cddis.2013.105 PMC3641328

[b33] Chatterjee B. . Developmental regulation and expression of the zebrafish connexin43 gene. Dev. Dyn. 233, 890–906 (2005). 15895415 10.1002/dvdy.20426

[b34] Gros D. B. & Jongsma H. J. Connexins in mammalian heart function. Bioessays 18, 719–730 (1996). 8831288 10.1002/bies.950180907

[b35] Lerner D. L., Yamada K. A., Schuessler R. B. & Saffitz J. E. Accelerated onset and increased incidence of ventricular arrhythmias induced by ischemia in Cx43-deficient mice. Circulation 101, 547–552 (2000). 10662753 10.1161/01.cir.101.5.547

[b36] Danik S. B. . Modulation of cardiac gap junction expression and arrhythmic susceptibility. Circ. Res. 95, 1035–1041 (2004). 15499029 10.1161/01.RES.0000148664.33695.2a PMC2956442

