# Supplementary material for: LRP6 acts as a scaffold protein in cardiac gap junction assembly
Source: Nat Commun. 2016 Jun 2;7:11775. doi: 10.1038/ncomms11775 (PMC4895718; doi:10.1038/ncomms11775)
Supplement: Supplementary Information — Supplementary Figures 1 - 12 and Supplementary Tables 1 - 3 [file ncomms11775-s1.pdf]

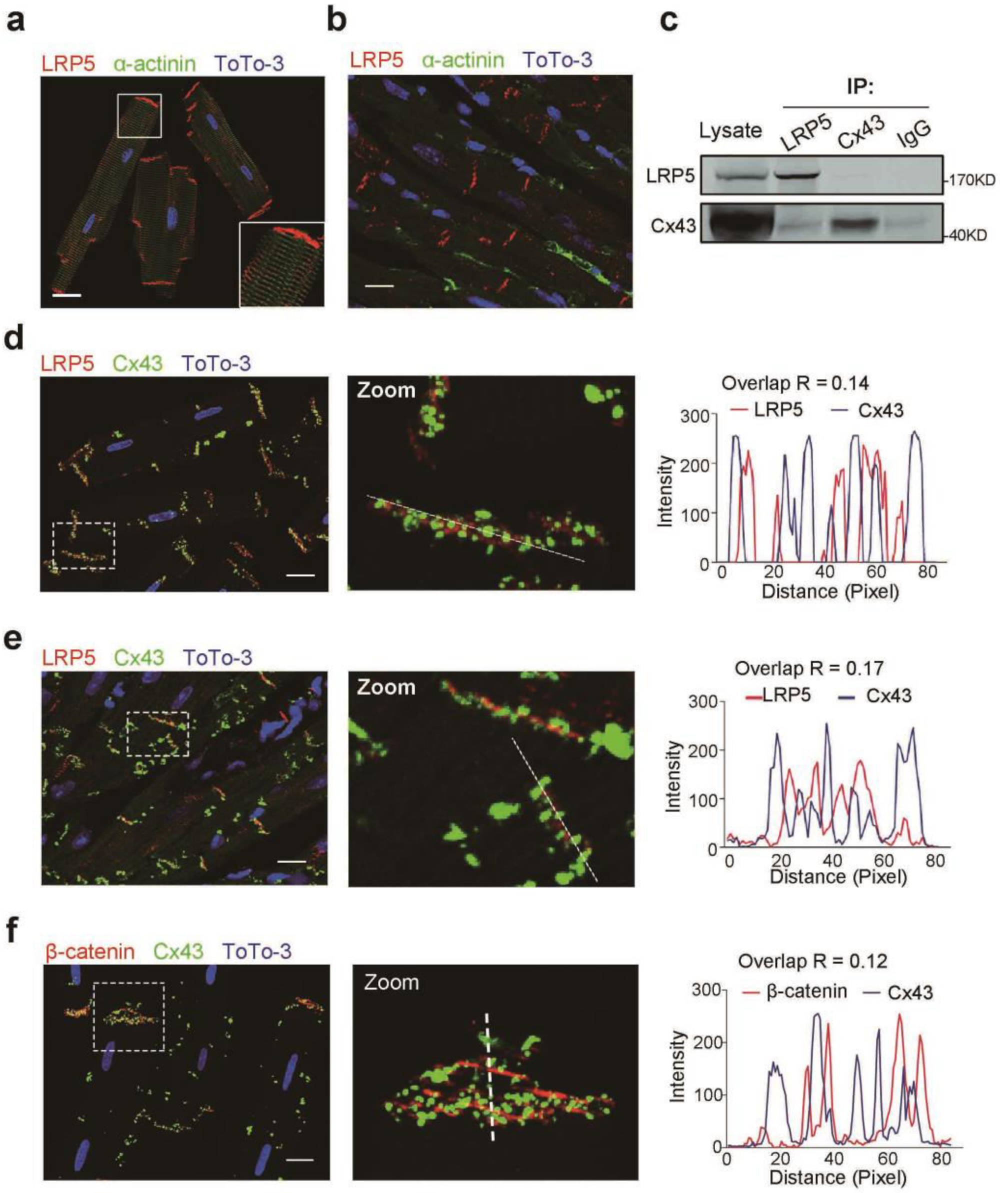

Supplementary Figure 1. Spatial distribution of LRP5 and β-catenin in intact cardiomyocytes. (**a**) and (**b**) Immunofluorescence staining of endogenous LRP5 in intact adult mouse ventricular myocytes (AMVMs) (**a**) and intact adult mouse ventricular tissues (**b**). Scale bars, 25 μm. Magnified images indicate the distribution patterns of LRP5 in the white boxes. (**c**) Co-immunoprecipitation of endogenous LRP5 and Cx43 in intact AMVMs. (**d**) and (**e**) Immunofluorescence labeling of endogenous LRP5 and connexin 43 (Cx43) in intact AMVMs (**d**) and intact adult mouse ventricular tissues (**e**). Scale bars, 25 μm. Magnified images indicating colocalization of LRP5 with Cx43 are shown in the white boxes. The right row indicates Pearson's correlation coefficient (R) and line plot profiles. (**f**) Immunofluorescence labeling of endogenous β-catenin and Cx43 in intact AMVMs. The right row in **d-f** indicates Pearson's correlation coefficient (R) and line plot profiles. Scale bars, 25 μm. The presented images and blots are representative of three separate experiments.

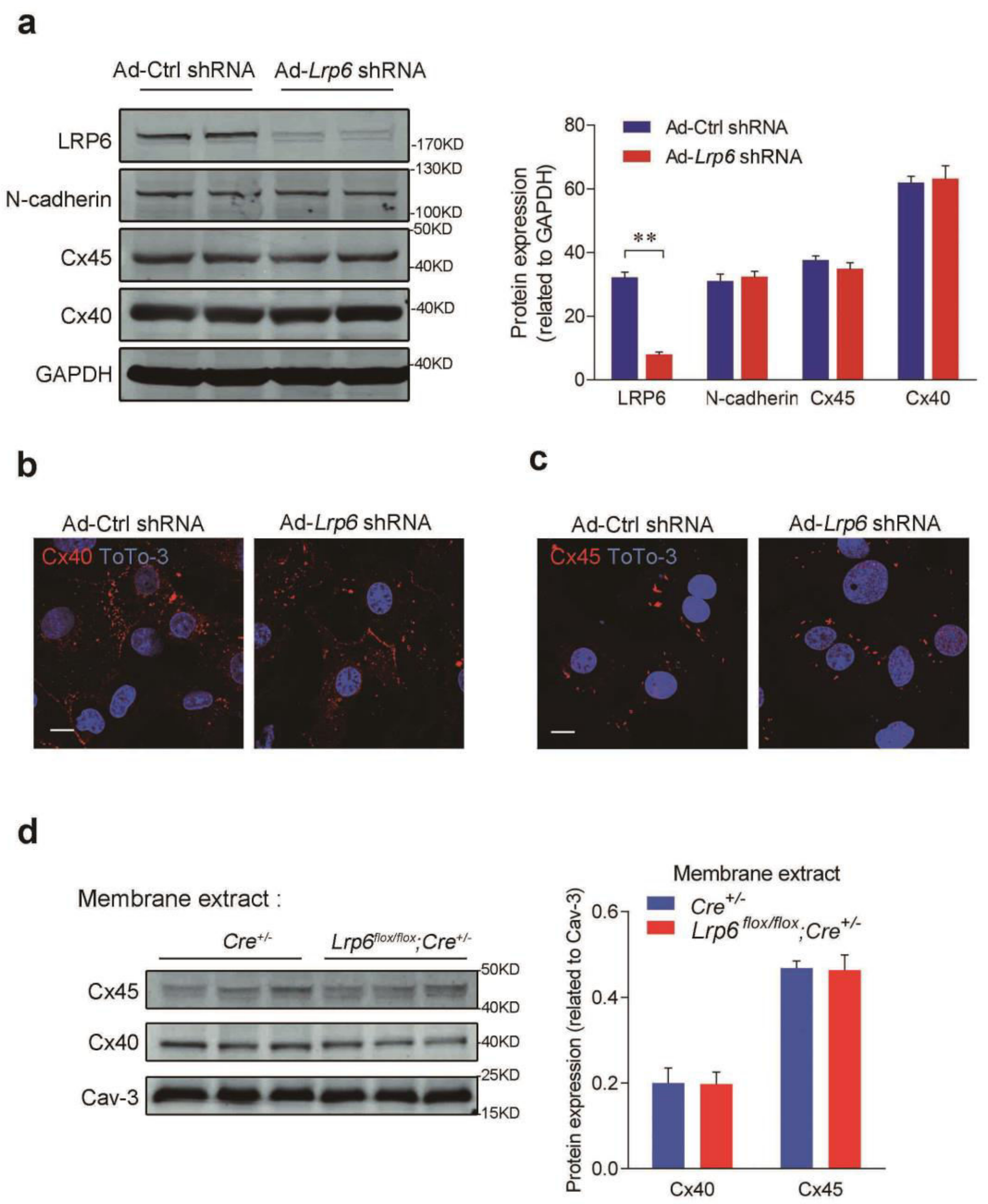

Supplementary Figure 2. Effects of LRP6 on Cx40, 45 and N-cadherin in cardiomyocytes. (**a**) Western blotting examination of Cx40, 45 and N-cadherin protein expression**.** *Left***,** typical western blots; right, pooled data. *n*=3. Data are means ± s.e.m. $^{**}P<0.01$ compared with ctrl by one-way ANOVA with Bonferroni's post hoc test. *n* represents the number of experiments. (**b**) and (**c**) Immunofluorescent imaging of Cx40 and Cx45 in LRP6-deficient NRVMs. Scale bars, 10 μm. The presented images are representative of three separate experiments. (**d**) Membrane expression of Cx40 and Cx45 in *Lrp6* knockout mice hearts. *Left***,** typical western blots; right, pooled data. Cav-3, caveolin 3, a cell membrane marker. *n*=3. Data are means ± s.e.m. *n* represents the number of experiments.

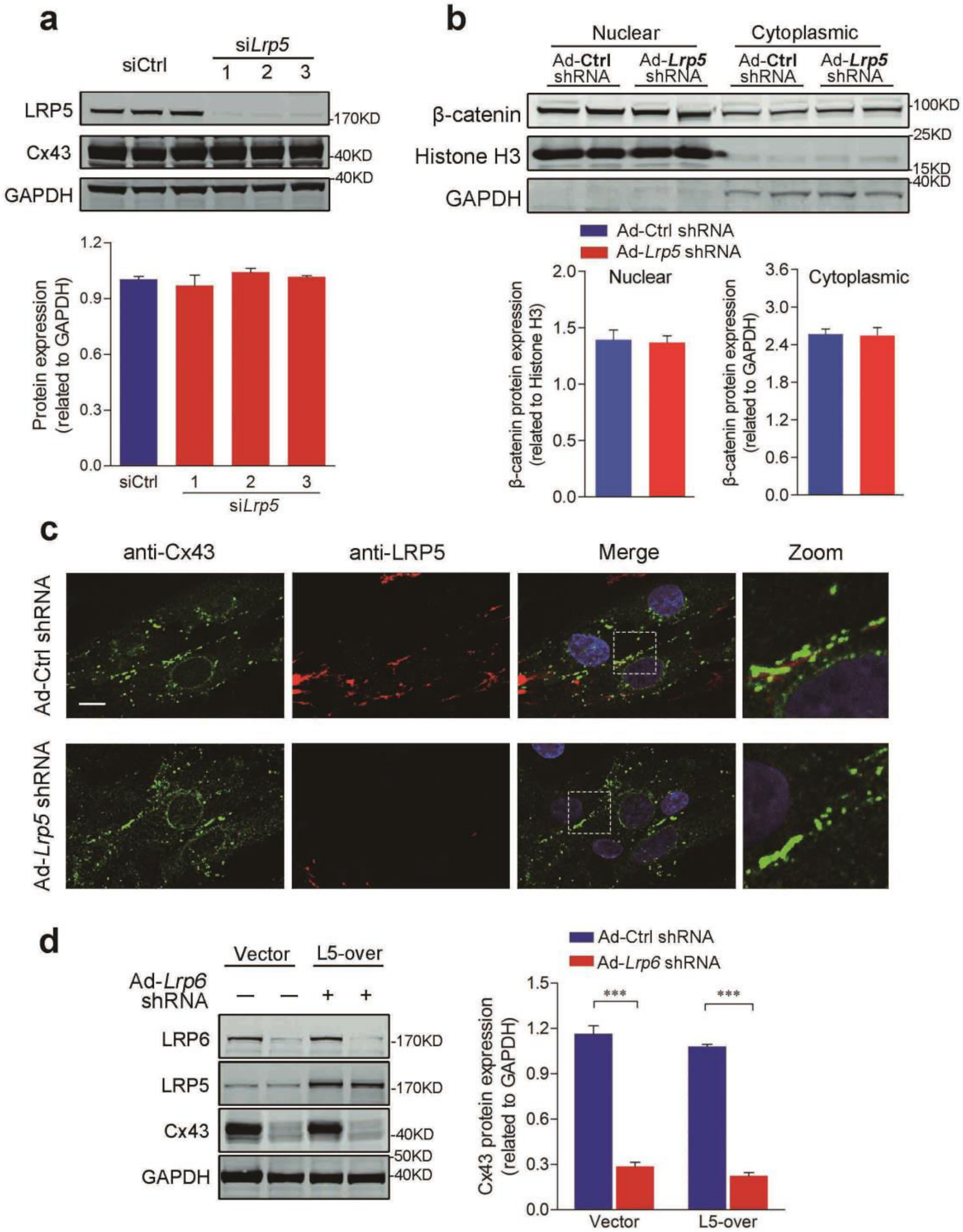
a
siCtrl
siLrp5
1 2 3
LRP5
-170KD
Cx43
-40KD
-40KD
GAPDH
Protein expression
(related to GAPDH)
1.2
0.9
0.6
0.3
0.0
siCtrl
1
2
3
siLrp5
b
Nuclear
Cytoplasmic
Ad-Ctrl shRNA
Ad-Lrp5 shRNA
Ad-Ctrl shRNA
Ad-Lrp5 shRNA
β-catenin
-100KD
-25KD
Histone H3
-15KD
-40KD
GAPDH
Ad-Ctrl shRNA
Ad-Lrp5 shRNA
Nuclear
β-catenin protein expression
(related to Histone H3)
2.0
1.5
1.0
0.5
0.0
Cytoplasmic
β-catenin protein expression
(related to GAPDH)
3.6
3.0
2.4
1.8
1.2
0.6
0.0
c
anti-Cx43
anti-LRP5
Merge
Zoom
Ad-Ctrl shRNA
Ad-Lrp5 shRNA
d
Vector
L5-over
Ad-Lrp6 shRNA
— — + +
LRP6
-170KD
LRP5
-170KD
Cx43
-40KD
-50KD
GAPDH
-40KD
Ad-Ctrl shRNA
Ad-Lrp6 shRNA
Cx43 protein expression
(related to GAPDH)
1.5
1.2
0.9
0.6
0.3
0.0
***
***
Vector
L5-over

Supplementary Figure 3. The deficiency of LRP5 does not depress the expression of Cx43. (**a**) Effects of *Lrp5* knockdown on Cx43 protein levels in cultured neonatal rat ventricular myocytes (NRVMs). *Top*, typical Western blots; *bottom*, pooled data. Three independent *Lrp5* siRNA sequences were employed. n=3. Data are means ± s.e.m. n represents the number of experiments. (**b**) Effects of LRP5 deficiency on Wnt signaling. The nuclear distribution of β-catenin was used for evaluating Wnt signaling activity. *Lrp5* knockdown did not change the nuclear expression of β-catenin. *Top*, typical Western blots; *bottom*, pooled data. n=3. Data are means ± s.e.m. n represents the number of experiments. (**c**) Immunofluorescence imaging of endogenous Cx43 in LRP5-deficient myocytes. Scale bars, 10 μm. The presented images are representative of three separate experiments. (**d**) Western blotting examination of Cx43 protein in LRP6-deficient NRVMs with or without LRP5 overexpression (L5-over). *Left*, typical blots; *right*, pooled data. n=3. Data are means ± s.e.m. ***P<0.001 compared with ctrl by one-way ANOVA with Bonferroni's post hoc test. n represents the number of experiments.

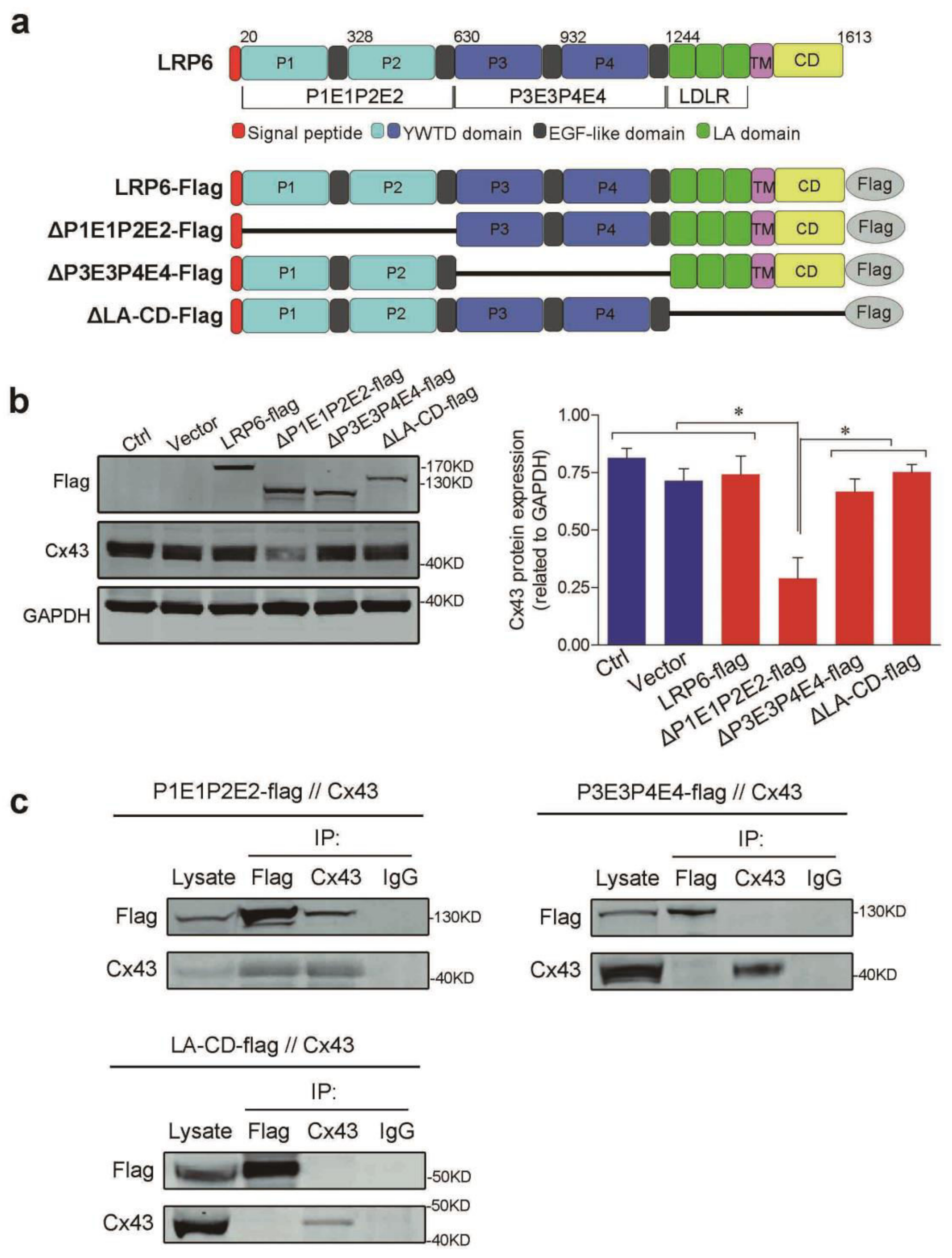

Supplementary Figure 4. Identification of the critical LRP6 module important for Cx43 expression. (**a**) The schematic diagram for LRP6 domain-truncated mutants. (**b**) Effects of LRP6 domain-truncated mutants on Cx43 protein expression. *Left*, typical western blots; *right*, pooled data. n=3. Data are means ± s.e.m. *P<0.05 compared with ΔP1E1P2E2-flag by one-way ANOVA with Bonferroni's post hoc test. n represents the number of experiments. (**c**) Co-immunoprecipitation examination of the interaction between LRP6 domain-truncated mutants and Cx43.

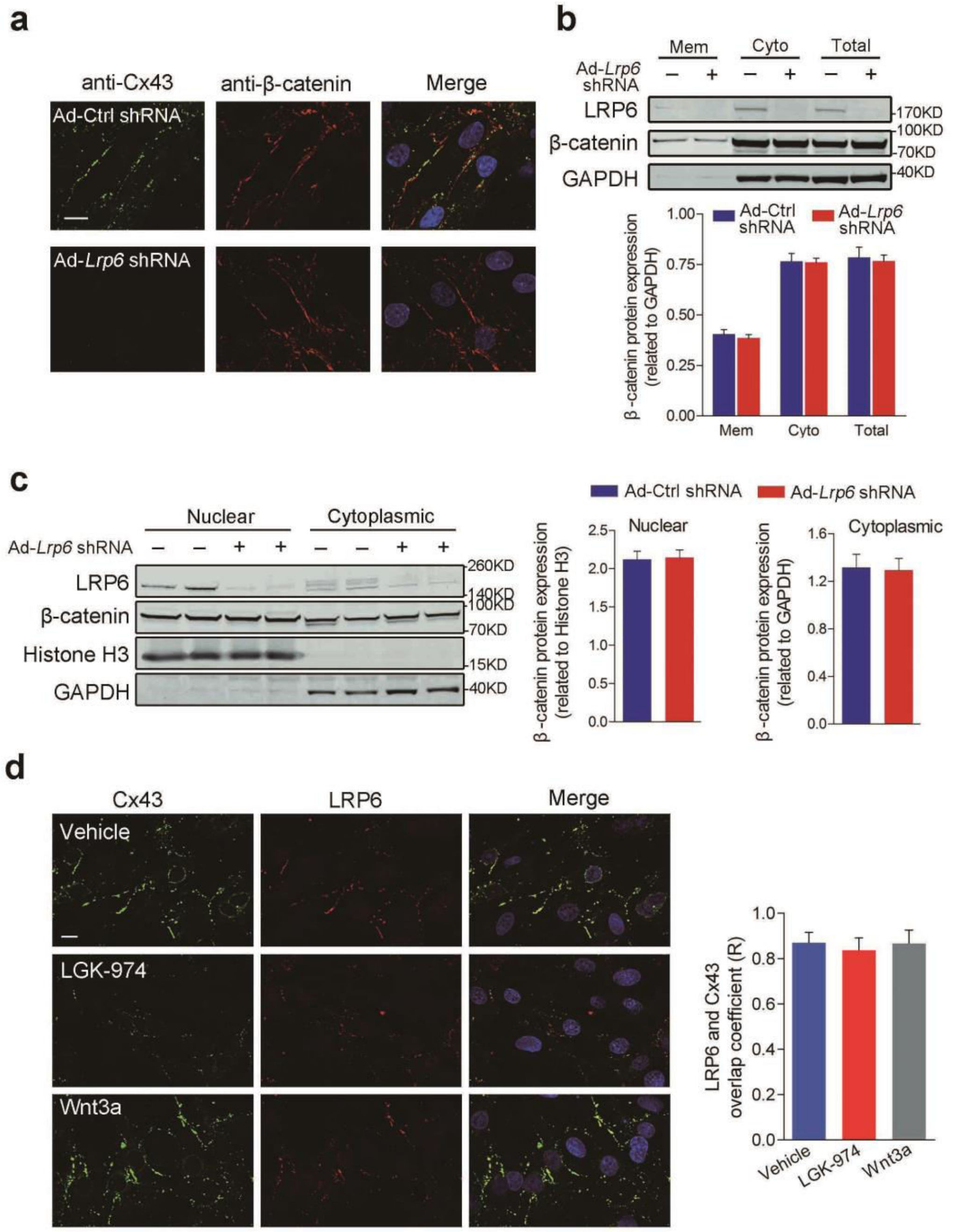

Supplementary Figure 5. Role of LRP6 in the canonical Wnt signaling of cardiomyocytes. (**a**) Immunofluorescent imaging of β-catenin and Cx43 in LRP6-deficient NRVMs. Scale bars, 10 µm. The presented images are representative of three separate experiments. (**b**) and (**c**) Western blotting examination of the fractional expression of β-catenin in LRP6-deficient NRVMs. Mem, membrane; Cyto, cytoplasmic. *n*=3. Data are means ± s.e.m. *n* represents the number of experiments. (**d**) Immunofluorescent imaging of LRP6 and Cx43 in NRVMs subject to Wnt activation and inhibition. LGK-974: Wnt inhibitor; Wnt3a: Wnt ligand. Scale bars, 10 µm. The presented images are representative of three separate experiments. *n*=3. Data are means ± s.e.m. *n* represents the number of experiments.

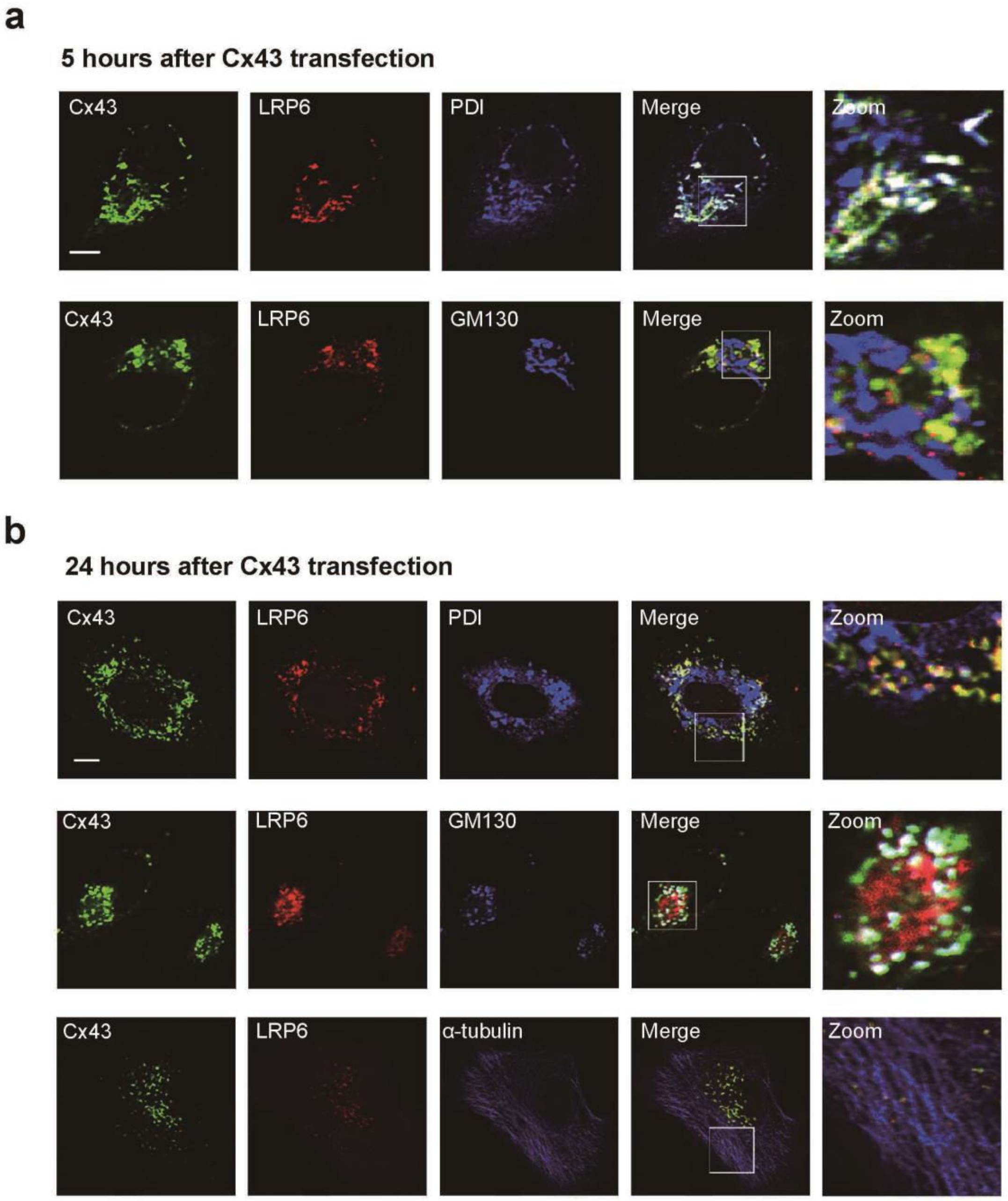

Supplementary Figure 6. Subcellular colocalization analysis of LRP6 and Cx43. The HeLa cells transfected with Cx43-GFP were used for cellular imaging. The colocalization of LRP6 and Cx43 was mostly observed in the ER (a triple colocalization of LRP6, Cx43 and PDI) at 5 hours post transfection (**a**), whereas their colocalization mostly occurred at the Golgi apparatus and along the microtubules at 24 hours post transfection as demonstrated by the triple colocalizations of LRP6/Cx43 with GM130 or α-tubulin (**b**). Scale bar, 10 μm. The presented images are representative of three separate experiments.

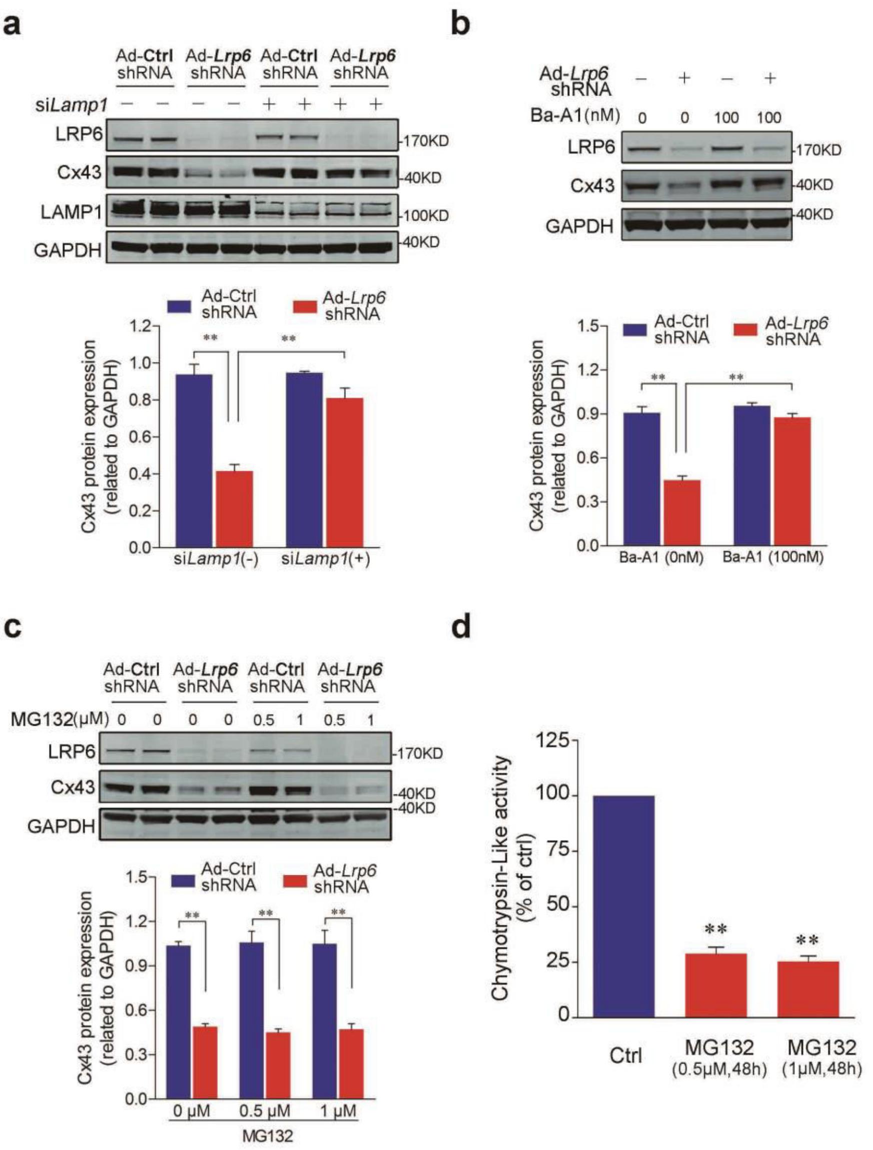

Supplementary Figure 7. LRP6 modulates the lysosomal degradation of Cx43 protein. (**a**) and (**b**) Western blotting analysis of the effects of lysosomal inhibition on Cx43 protein expression in LRP6-deficient NRVMs. *Lamp1* siRNA and Ba-A1 (Bafilomycin A1) were adopted to inhibit lysosomal degradation. *n*=3 (a) and (b). Data are means ± s.e.m. ***P*<0.01 compared with ctrl by one-way ANOVA with Bonferroni's post hoc test. *n* represents the number of experiments. (**c**) Effects of a proteolytic inhibitor of the 26S proteasome complex, MG132, on Cx43 protein in LRP6-deficient cardiomyocytes. The immunoblots show that proteasomal inhibition did not counteract the reduction in the Cx43 protein level by *Lrp6* knockdown. *n*=3. Data are means ± s.e.m. ***P*<0.01 compared with ctrl by one-way ANOVA with Bonferroni's post hoc test. *n* represents the number of experiments. (**d**) Assay of chymotrypsin-like activity of the proteasome. Treatment with 0.5-1 mM MG132 for 48 hours significantly inhibited the chymotrypsin-like activity of the proteasome in NRVMs. *n*=3. Data are means ± s.e.m. ***P*<0.01 compared with ctrl by one-way ANOVA with Bonferroni's post hoc test. *n* represents the number of experiments.

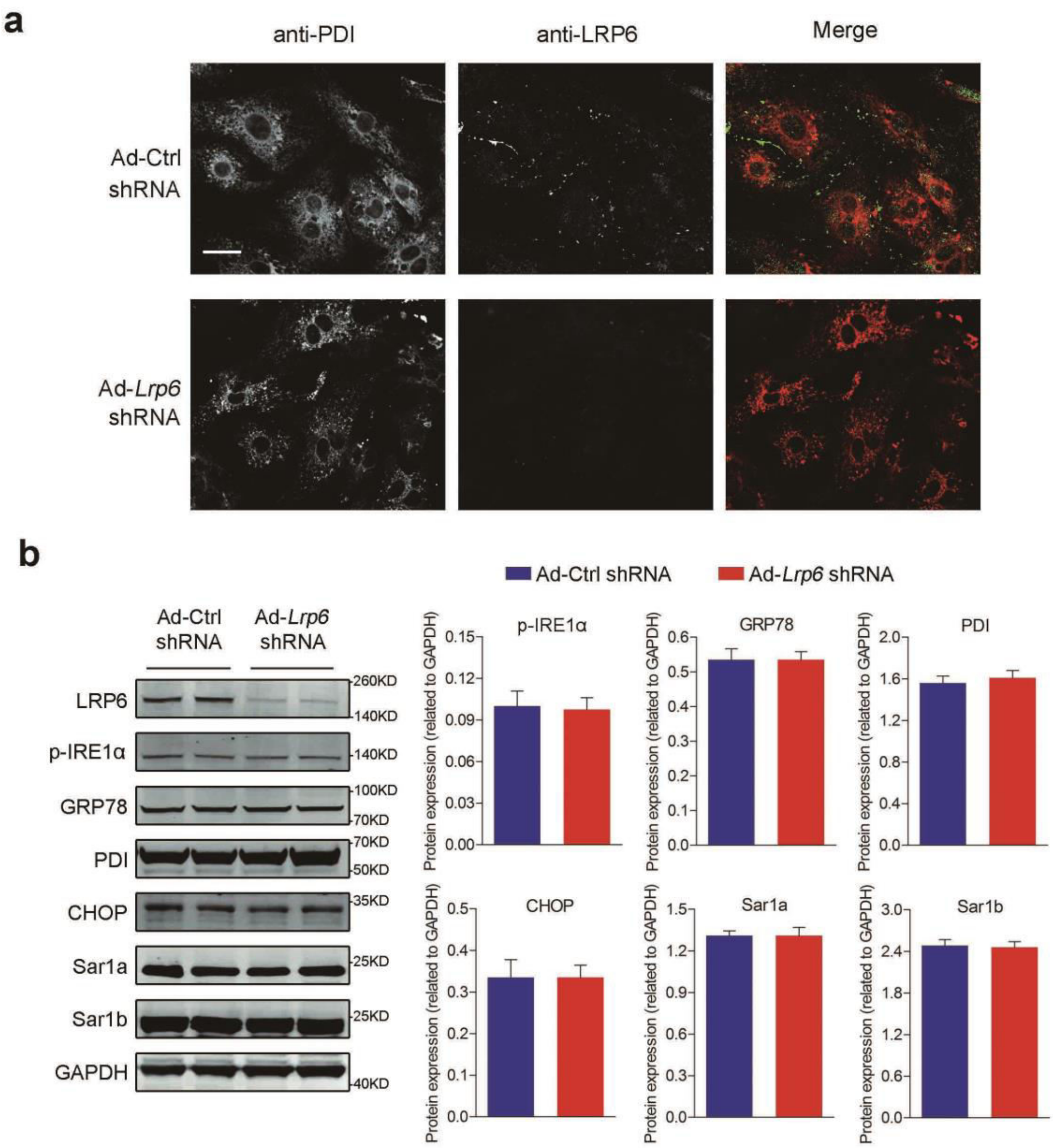

Supplementary Figure 8. Effects of LRP6 reduction on the ER morphology and function. (**a**) Immunofluorescent imaging of ER in LRP6-deficient cardiomyocytes. PDI: a ER marker. Scale bars, 10 μm. The presented images are representative of three separate experiments. (**b**) Western blotting examination of the effects of LRP6 on the molecules for ER stress response, chaperons and protein exit machinery. *Left*, typical blots; right: pooled data. *n*=3. Data are means ± s.e.m. *n* represents the number of experiments.

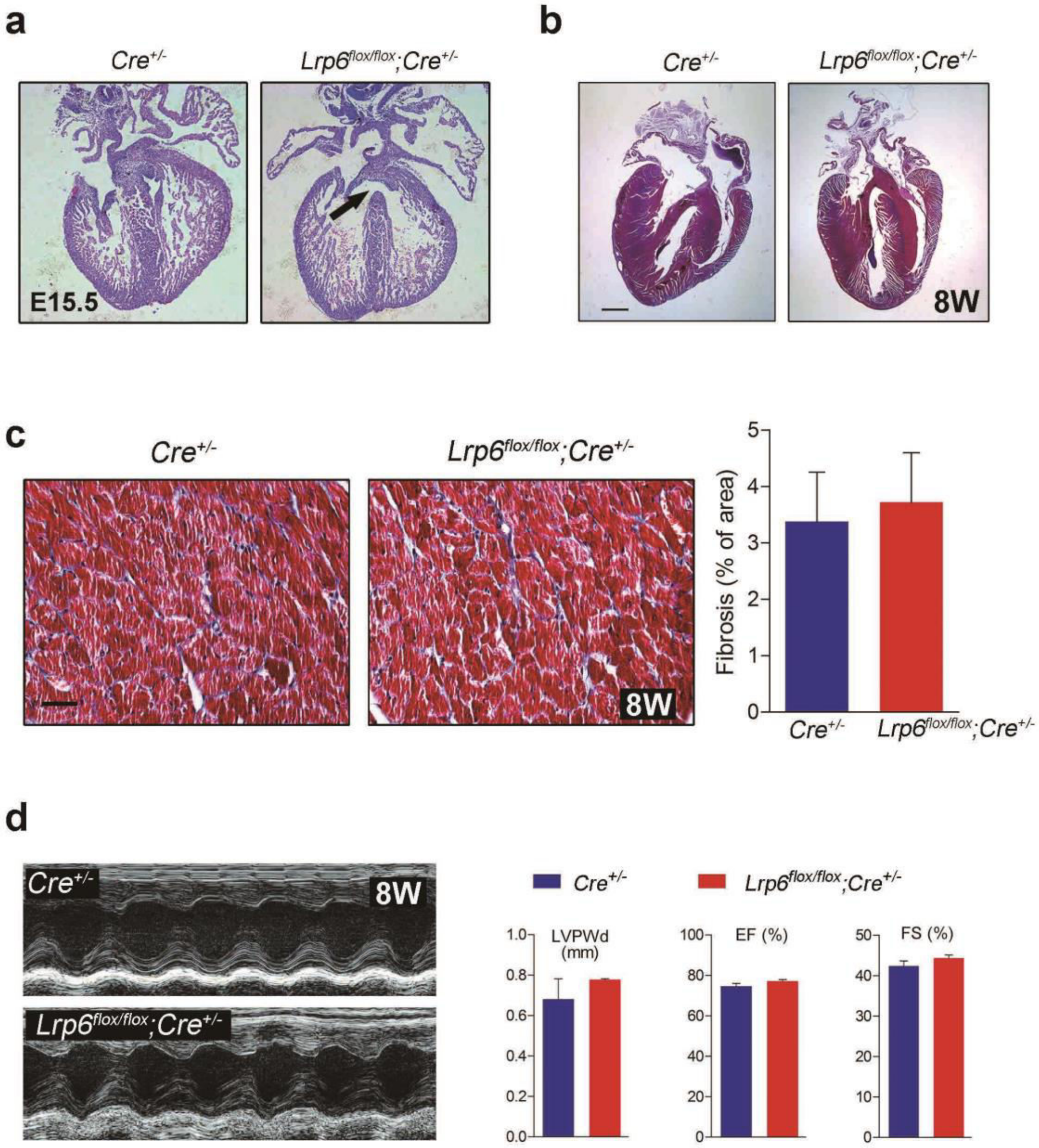

Supplementary Figure 9. Effects of *Lrp6* CKO on cardiac structure and function. (**a**) and (**b**) Immunohistochemical analysis of the *Lrp6*-knockout embryonic and adult hearts. Scale bar, 1 mm. (**c**) Histological analysis of collagen throughout the myocardium. Fibrosis was determined as the ratio of the area of fibrosis to the total vessel area by using ImageJ software. Scale bar, 100 µm. n=5. Data are means ± s.e.m. n represents the number of experiments. (**d**) Echocardiographic measurements of cardiac function. *Left*, representative echocardiograms; *right*, pooled data. There are no significant alterations in LV ejection fraction (EF) or fractional shortening (FS) or LV posterior wall dimensions (LVPWd) in the cardiac-specific *Lrp6*-knockout mice. n=5. Data are means ± s.e.m. n represents the number of experiments.

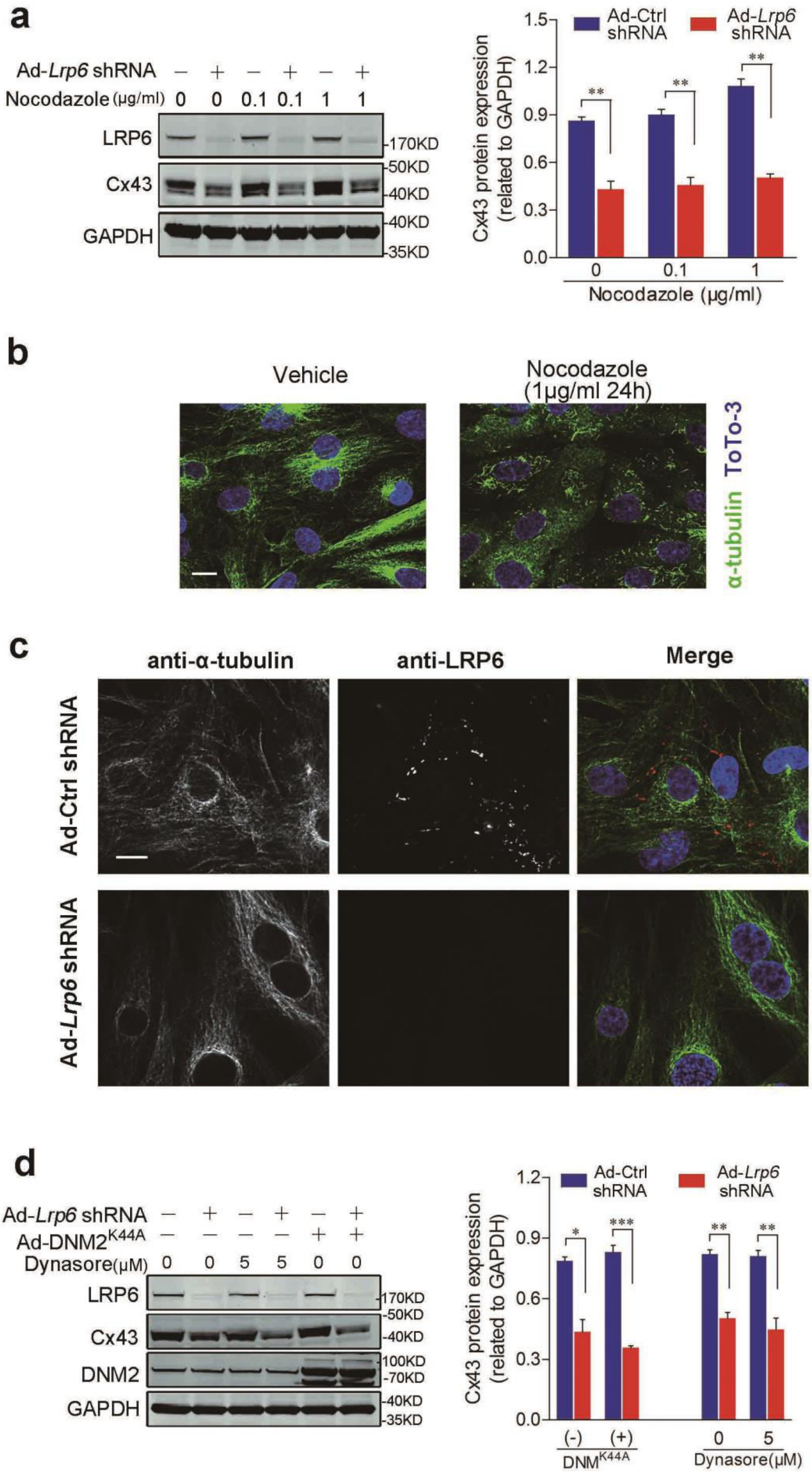
a
Ad-Lrp6 shRNA
Nocodazole (μg/ml)
0 0 0.1 0.1 1 1
LRP6
Cx43
GAPDH
170KD
50KD
40KD
40KD
35KD
Ad-Ctrl shRNA
Ad-Lrp6 shRNA
Cx43 protein expression (related to GAPDH)
1.5
1.2
0.9
0.6
0.3
0.0
**
0
0.1
1
Nocodazole (μg/ml)
b
Vehicle
Nocodazole (1μg/ml 24h)
ToTo-3
α-tubulin
c
anti-α-tubulin
anti-LRP6
Merge
Ad-Ctrl shRNA
Ad-Lrp6 shRNA
d
Ad-Lrp6 shRNA
Ad-DNM2K44A
Dynasore(μM)
0 0 5 5 0 0
LRP6
Cx43
DNM2
GAPDH
170KD
50KD
40KD
100KD
70KD
40KD
35KD
Ad-Ctrl shRNA
Ad-Lrp6 shRNA
Cx43 protein expression (related to GAPDH)
1.2
0.9
0.6
0.3
0.0
*
***
**
(-)
(+)
DNMK44A
0
5
Dynasore(μM)

Supplementary Figure 10. *Lrp6* knockdown does not affect the membrane trafficking of Cx43. (**a**) Western blotting analysis of Cx43 protein in cardiomyocytes subjected to nocodazole treatment or *Lrp6* knockdown. *Left*, typical blots; *right*, pooled data. n=3. Data are means ± s.e.m. **P<0.01 compared with ctrl by one-way ANOVA with Bonferroni's post hoc test. n represents the number of experiments. (**b**) Immunofluorescence staining of α-tubulin in NRVMs under nocodazole treatment for 24 h. Scale bar, 10 μm. (**c**) Immunofluorescence of labeled α-tubulin and LRP6 in NRVMs following *Lrp6* knockdown. ToTo-3 indicated the cell nucleus. Scale bar, 10 μm. The presented images are representative of three separate experiments. (**d**) The immunoblots demonstrates the protein expression of Cx43 in LRP6-deficient cardiomyocytes subjected to dynamin activity inhibition. Dynasore (an inhibitor of dynamin) treatment and overexpression of $DNM2^{K44A}$ (a dominant negative dynamin-2 mutant) were adopted to inhibit dynamin activity in the cardiomyocytes. n=3. Data are means ± s.e.m. **P<0.01 compared with ctrl by one-way ANOVA with Bonferroni's post hoc test. n represents the number of experiments.

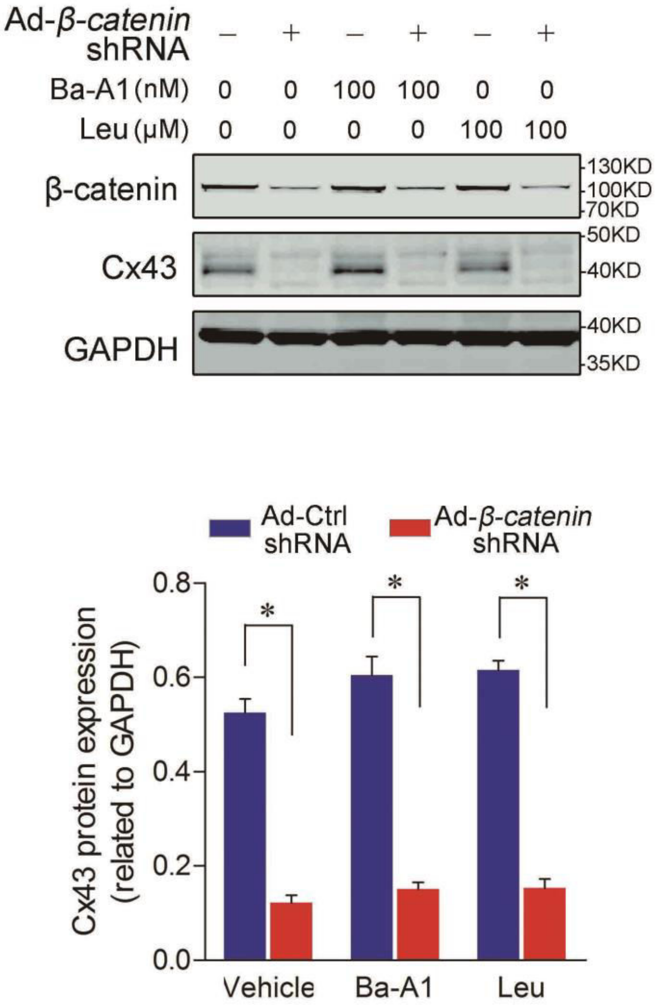

Supplementary Figure 11. Reduced Cx43 by *β-catenin* knockdown is not rescued by Leu and Ba-A1. *Top*, Representative western blots for Cx43 and β-catenin. Leupeptin: Leu; Bafilomycin A1: Ba-A1. The presented blots are representative of three separate experiments. Bottom, pooled data. *n*=3. Data are means ± s.e.m. **P*<0.05 compared with ctrl by one-way ANOVA with Bonferroni's post hoc test. *n* represents the number of experiments.

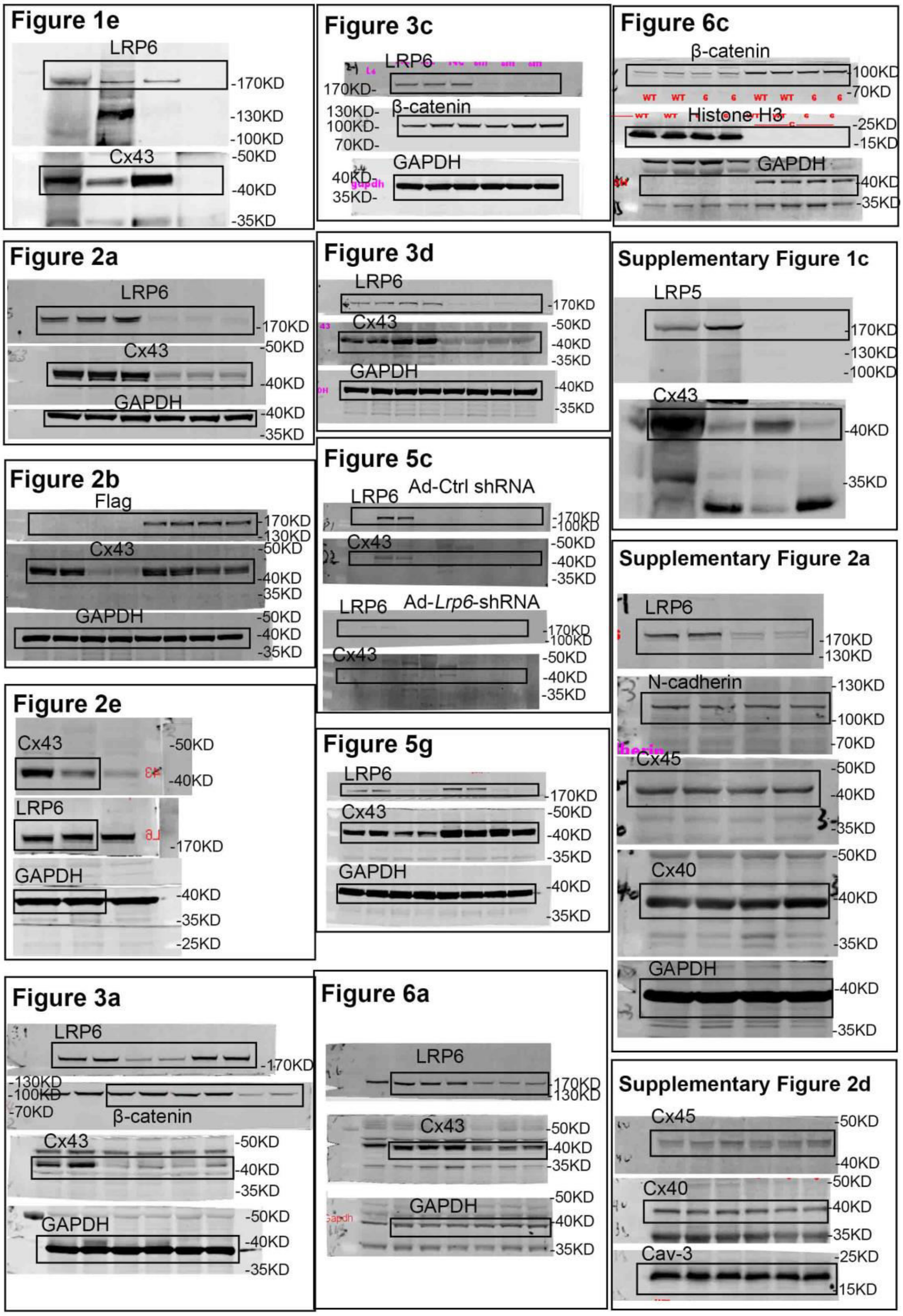

Figure 1e
LRP6
-170KD
-130KD
-100KD
Cx43
-50KD
-40KD
-35KD
Figure 2a
LRP6
-170KD
Cx43
-50KD
-40KD
GAPDH
-40KD
-35KD
Figure 2b
Flag
-170KD
-130KD
Cx43
-50KD
-40KD
-35KD
GAPDH
-50KD
-40KD
-35KD
Figure 2e
Cx43
-50KD
-40KD
LRP6
-170KD
GAPDH
-40KD
-35KD
-25KD
Figure 3a
LRP6
-170KD
-130KD
-100KD
-70KD
β-catenin
Cx43
-50KD
-40KD
-35KD
GAPDH
-50KD
-40KD
-35KD
Figure 3c
LRP6
170KD-
130KD-
β-catenin
100KD-
70KD-
GAPDH
40KD-
35KD-
Figure 3d
LRP6
-170KD
Cx43
-50KD
-40KD
-35KD
GAPDH
-40KD
-35KD
Figure 5c
LRP6
Ad-Ctrl shRNA
-170KD
-100KD
Cx43
-50KD
-40KD
-35KD
LRP6
Ad-Lrp6-shRNA
-170KD
-100KD
Cx43
-50KD
-40KD
-35KD
Figure 5g
LRP6
-170KD
Cx43
-50KD
-40KD
-35KD
GAPDH
-40KD
-35KD
Figure 6a
LRP6
-170KD
-130KD
Cx43
-50KD
-40KD
-35KD
GAPDH
-50KD
-40KD
-35KD
Figure 6c
β-catenin
-100KD
-70KD
Histone H3
-25KD
-15KD
GAPDH
-40KD
-35KD
Supplementary Figure 1c
LRP5
-170KD
-130KD
-100KD
Cx43
-40KD
-35KD
Supplementary Figure 2a
LRP6
-170KD
-130KD
N-cadherin
-130KD
-100KD
-70KD
Cx45
-50KD
-40KD
-35KD
Cx40
-50KD
-40KD
-35KD
GAPDH
-40KD
-35KD
Supplementary Figure 2d
Cx45
-50KD
-40KD
Cx40
-50KD
-40KD
-35KD
Cav-3
-25KD
-15KD

Supplementary Figure 3a

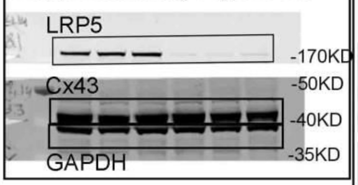

Supplementary Figure 3b

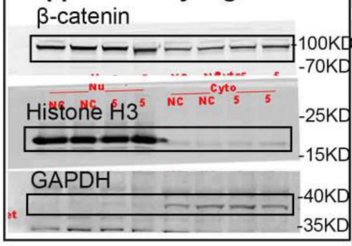

Supplementary Figure 3d

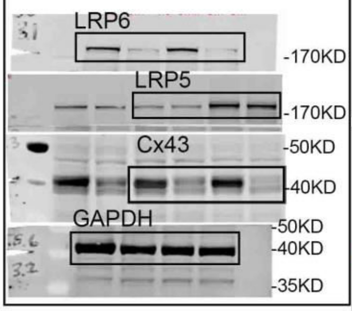

Supplementary Figure 4b

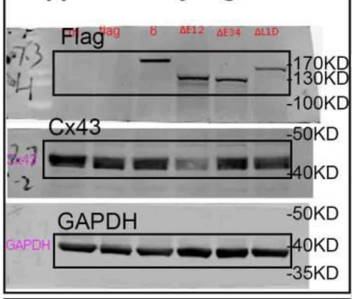

Supplementary Figure 4c

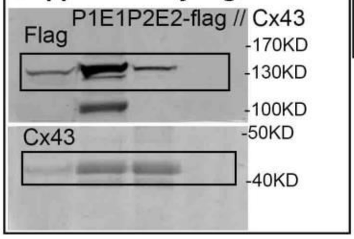

Supplementary Figure 4c

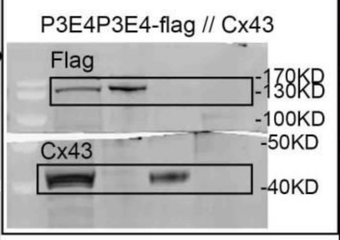

Supplementary Figure 4c

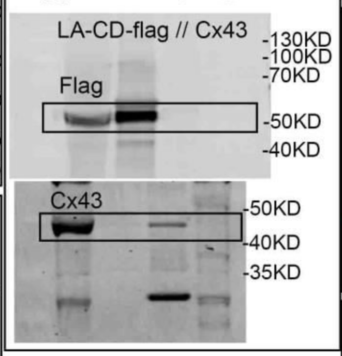

Supplementary Figure 5b

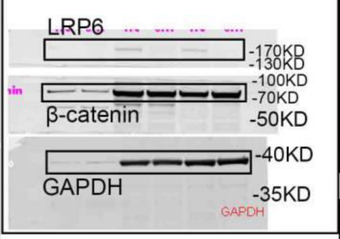

Supplementary Figure 5c

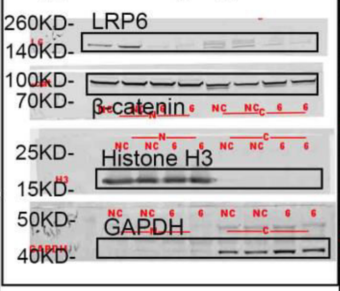

Supplementary Figure 7a

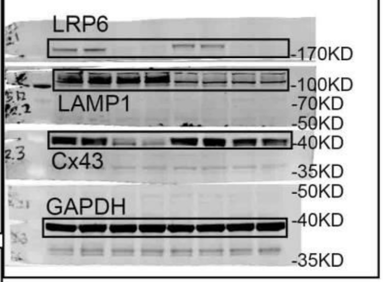

Supplementary Figure 7b

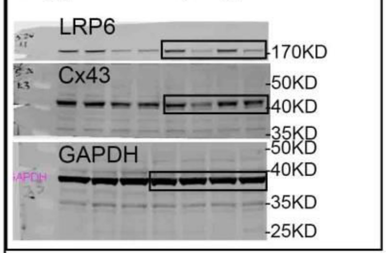

Supplementary Figure 7c

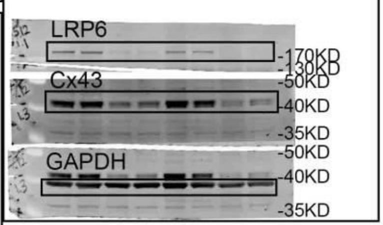

Supplementary Figure 8b

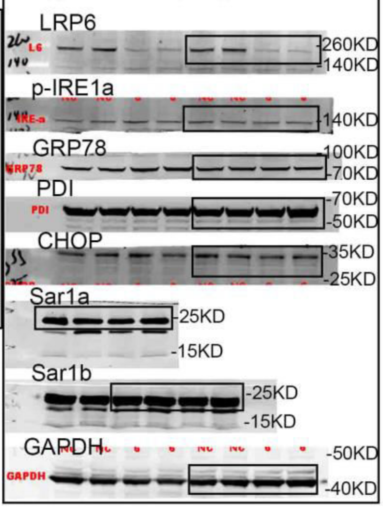

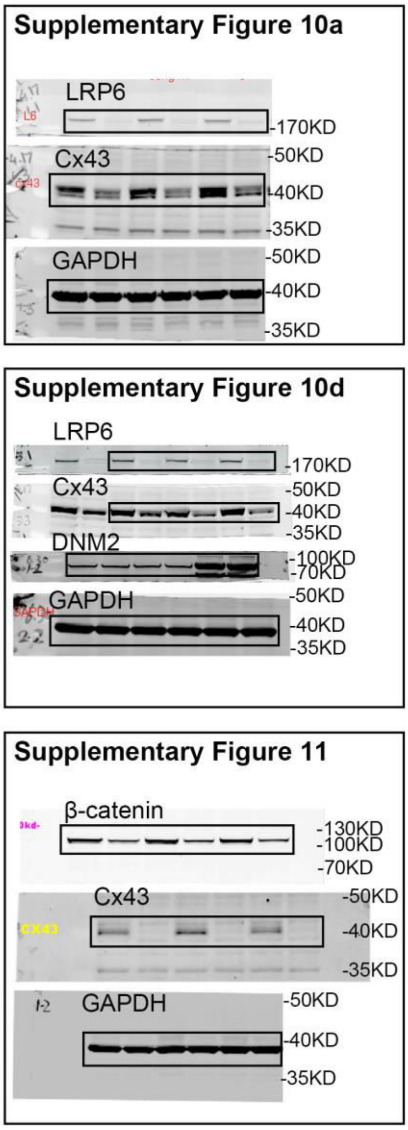

Supplementary Figure 12. Full scans of western blots data. Black boxes highlight the indicated lanes in figures.

Supplementary Table 1. Primer sequences

Rat-18s-Forward Primer	TGCGGAAGGATCATTAACGGA
Rat-18s-Reverse Primer	AGTAGGAGAGGAGCGAGCGACC
Rat-LRP6-Forward Primer	GCAGTGAAGGAGCTGAACCT
Rat-LRP6-Reverse Primer	CCTGTAGGATGGTGGTGGGT
Rat-Cx43-Forward Primer	AGGCGTGAGGAAAGTACCAAA
Rat-Cx43-Reverse Primer	CCGTGCTCTTCAATCCCGTA
Rat-β-catenin-Forward Primer	CAGTGCAGGAGGCCGAG
Rat-β-catenin-Reverse Primer	GGCCATGTCCAACTCCATCA
Mouse-Lrp6-Forward Primer	CTGGATTATTGTCCCCGGAT
Mouse-Lrp6-Reverse Primer	GTGTGTATTCCAGTCAGTCC
Mouse-Cx43-Forward Primer	AAAGGCGTGAGGGAAGTACC
Mouse-Cx43-Reverse Primer	ACAGCGAAAGGCAGACTGTT
Mouse-GAPDH-Forward Primer	GGTGAAGGTCGGTGTGAACG
Mouse-GAPDH-Reverse Primer	CTCGCTCCTGGAAGATGGTG

Supplementary Table 2. shRNAs sequences

Lrp6	GGCAGACACAGGAACAAAU
Lrp5	GGCUCUGGAUCCUACUAAA
Cx43	CAAUUCCUCGUGCCGCAA
β-catenin	GAGACUGCCUUCAGAUCUU

Supplementary Table 3. siRNAs sequences

Lrp6	GGCAGACACAGGAACAAAU
	CCAUGGAUAUACAUGCUUU
	GCAUUGUUCCUGAGGCUUU
Lrp5	GGCUCUGGAUCCUACUAAA
	CCUGGAGACUAACAACAAU
	CGAGGGUACAUGUACUUUA
Lamp1	GGGUCUACAUGAAGAAUGU
